# Glutamatergic and GABAergic neurons in the preoptic area of the hypothalamus play key roles in menopausal hot flashes

**DOI:** 10.3389/fnagi.2022.993955

**Published:** 2022-10-14

**Authors:** Yanrong Sun, Hanfei Wang, Wenjuan Wang, Jiali Lu, Jinglin Zhang, Xiaofeng Luo, Liju Luan, Ke Wang, Jing Jia, Junhao Yan, Lihua Qin

**Affiliations:** ^1^Department of Human Anatomy, Histology and Embryology, School of Basic Medical Sciences, Peking University Health Science Center, Beijing, China; ^2^Department of Stomatology, Shanxi Medical University School and Hospital of Stomatology, Taiyuan, Shanxi, China; ^3^Department of Stomatology, The Third Medical Center, Chinese PLA General Hospital, Beijing, China; ^4^Beijing Key Lab of Magnetic Resonance Imaging Technology, Peking University Third Hospital, Beijing, China

**Keywords:** hot flashes, glutamatergic neurons, GABAergic neurons, glutamate decarboxylase, thermosensitive transient receptor potentials, estrogen receptors

## Abstract

During menopause, when estrogen levels are low, abnormalities in the hypothalamic preoptic area (POA) of the thermoregulatory center can cause hot flashes. However, the involved neural population has not been identified. Proteomics showed that under low estrogen, differentially expressed proteins in the hypothalamus were associated with glutamatergic and GABAergic synapses. RNAscope, Western blotting and qRT-PCR indicated that the number of glutamatergic neurons in the POA was decreased, while the number of GABAergic neurons was increased. Chemogenetics showed that the rat body temperature decreased slowly after glutamatergic neurons were activated and increased quickly after glutamatergic neurons were inhibited, while it increased quickly after GABAergic neurons were activated and decreased slowly after GABAergic neurons were inhibited. RNAscope, immunofluorescence, Western blotting and qRT-PCR further showed that glutamate decarboxylase (GAD) 1 expression in the POA was increased, while GAD2 expression in the POA was decreased; that thermosensitive transient receptor potential protein (ThermoTRP) M (TRPM) 2 expression in glutamatergic neurons was decreased, while TRPM8 expression in GABAergic neurons was increased; and that estrogen receptor (ER) α and β expression in the POA was decreased, and ERα and ERβ expressed in both glutamatergic and GABAergic neurons. Estrogen therapy corrected these abnormalities. In addition, CUT&Tag and Western blot after injection of agonists and inhibitors of ERs showed that ERα and ERβ were both transcription factors in glutamatergic and GABAergic synapses. Mechanistically, during menopause, estrogen may regulate the transcription and expression of GADs and ThermoTRPs through ERs, impacting the number and function of glutamatergic and GABAergic neurons, resulting in unbalanced heat dissipation and production in the POA and ultimately triggering hot flashes.

## Graphical Abstract

DEPs, differentially expressed proteins; ERα, estrogen receptor α; ERβ, estrogen receptor β; GAD1, glutamate decarboxylase 2; GAD2, glutamate decarboxylase 2; POA, preoptic area; TRPM2, transient receptor potential melastatin-related 2; TRPM8, transient receptor potential melastatin-related 8; Vgat, vesicular GABA transporter; Vglut2, vesicular glutamate transporter 2. 
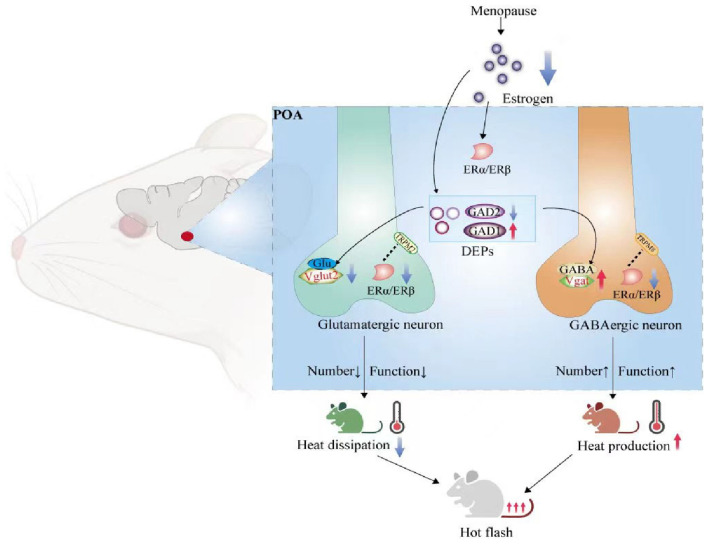

## Introduction

Hot flashes, which are common in menopausal women, are a characteristic indicator of ovarian dysfunction and a secondary factor associated with other menopausal symptoms after estrogen levels decrease (Stuenkel, 2018; Bansal and Aggarwal, 2019). Hot flashes are characterized by sudden strong fever or sweating on the face, neck and upper chest area, as well as palpitations, anxiety, irritability, and panic. In addition, the peripheral and central body temperatures change (Freedman et al., 1995; Freedman, 2000; Ryu et al., 2020). Hot flashes often occur at night, interfering with sleep, affecting mental health, and lowering quality of life (de Zambotti et al., 2014; Stuenkel, 2021). Previous studies have shown that up to 85% of women experience hot flashes. Hot flashes are associated with an increased risk of chronic diseases such as obesity, metabolic syndrome, insulin resistance, non-alcoholic fatty liver, cardiovascular disease, and osteoporosis (Lee et al., 2012; Kwon et al., 2016; Ryu et al., 2016, 2018), and they are regarded as an early warning sign of cardiovascular disease (Rathnayake et al., 2019; Ryu et al., 2020). However, the pathophysiology of hot flashes is unknown. Although estrogen replacement therapy is still the most effective treatment for hot flashes (MacLennan et al., 2002; Nelson, 2004; Chu et al., 2018), long-term use can increase the incidence of tumors in target organs (Couzin and Enserink, 2002; Marjoribanks et al., 2017; Ruan and Mueck, 2018). Other studies have reported that estrogen supplementation may increase the risk of stroke, diabetes and cholelithiasis (Couzin and Enserink, 2002; Turgeon et al., 2004; Langer, 2017). Because of the contraindications and side effects of estrogen therapy, the focus of research has shifted to identifying precise estrogen targets and treating them with non-hormonal drugs (such as neurotransmitter modulators).

Hot flashes are currently thought to be produced by abnormal function of the hypothalamic preoptic area (POA), a thermoregulatory center, caused by a decrease in estrogen levels (Freedman, 2014; Minkin, 2019). Estrogen has a considerable influence on autonomic thermoregulation. Human studies have shown that as the estrogen level of menopausal women decreases, core body temperature increases, and that oral estrogen treatment can restore estrogen levels (Freedman, 2014). Animal studies have revealed that ovariectomized rats have abnormal thermoregulatory function, increased peripheral and central body temperature, and significantly increased fluctuations in peripheral body temperature per unit time, and that estrogen could effectively improve the above abnormalities (Freedman and Dinsay, 2000; Ma et al., 2011; Sun et al., 2016). In the thermoregulatory loop, peripheral and central temperature information is transmitted to the hypothalamic POA; heat signals are transmitted to glutamatergic neurons, which are mediated by the excitatory neurotransmitter glutamate, while cold signals are transmitted to GABA neurons, which are mediated by the inhibitory neurotransmitter GABA. After the temperature signal is integrated and processed by the thermoregulatory center, an instruction signal is sent to the peripheral effector through the efferent nerve pathway and neuroendocrine pathway, allowing the body to maintain a relatively stable temperature when the external temperature changes (Nakamura, 2011; Nakamura and Morrison, 2011; Zhao et al., 2017). Thus, the thermoregulatory center, the hypothalamic POA, may regulate heat dissipation and production pathways mainly through glutamatergic and GABAergic neurons. However, it remains to be seen whether these two types of neurons change when estrogen levels are low, and whether this change leads to POA dysfunction in regulating heat dissipation and production, resulting in hot flashes.

The effect of estrogen on glutamate and GABA has not been reported in humans; however, it has been studied in animal models *in vitro*. Several studies have shown that estrogen enhances glutamatergic synaptic transmission in the hippocampus and reduces GABA-mediated signal transduction in the hippocampus, amygdala and midbrain (McGinnis et al., 1980; Febo and Segarra, 2004; Oberlander and Woolley, 2016). In addition, estrogen inhibited GABA input in cultured rat hippocampal neurons *in vitro* (Murphy et al., 1998). Compared with male rats not treated with estrogen, female rats and male rats treated with estrogen showed a significant increase in functional glutamatergic neurotransmission (Wei et al., 2014). *In vivo* studies on rats and baboons have also shown that estrogen reduces GABA activity (McGinnis et al., 1980; Dewey et al., 1992). Overall, the above results suggest that estrogen may have a regulatory effect on the glutamatergic and GABAergic pathways. Therefore, whether menopausal hot flashes are caused by abnormal glutamatergic and GABAergic neurons in the hypothalamic POA under low estrogen states, as well as how low estrogen states regulate glutamatergic and GABAergic neurons, need to be explored further.

Glutamate has a number of functions in the mammalian central nervous system. Glutamate is an excitatory neurotransmitter and a direct precursor of the inhibitory neurotransmitter GABA (Petroff, 2002; Winter and Ueda, 2008). Data have shown that glutamate decarboxylase (GAD), a key rate-limiting enzyme in the conversion of glutamate to GABA, is widely expressed in the brain (Saiz et al., 2008; Arafat et al., 2009; Niu et al., 2009). There are two GAD isomers in the brain, GAD1 (GAD67) and GAD2 (GAD65), with relative molecular weights of approximately 67000 and 65000, respectively (Martyniuk et al., 2007). Previous studies have shown that GAD1 and GAD2 synthesize more than 99% of GABA in the brain (Ji et al., 1999; Ottem et al., 2004). In ovariectomized rats treated with estrogen, GAD1 and GAD2 mRNA levels in the periventricular nucleus of the forebrain decreased and increased, respectively (Bosma et al., 2001; Curran-Rauhut and Petersen, 2002), suggesting that estrogen may regulate the expression of GAD1 and GAD2. Furthermore, data have shown that the estrogen nuclear receptors ERα and ERβ mediate the “genotype regulation effect” of estrogen, which occurs when estrogen binds closely to a nuclear receptor after entering a cell and regulates gene transcription and expression by binding to the estrogen response element (ERE) on the target gene (Krolick et al., 2018). In addition, a double luciferase assay was used to identify three EREs in the pre-3000 bp upstream of the transcription start sites (TSSs) of the GAD2 promoter in rats (Pedersen et al., 2001; Hudgens Ji et al., 2009). Therefore, we speculate that estrogen may regulate the transcription and expression of GAD1 and GAD2 in the POA through its nuclear receptors, affecting the synthesis and release of glutamate and GABA and thus leading to changes in the number of glutamatergic and GABAergic neurons.

The hypothalamic POA, which serves as the thermoregulatory center in the brain, uses the activity of thermosensitive neurons to regulate body temperature, while the functional activity of thermosensitive neurons depends on the activation of their thermosensitive ion channels (Diaz-Franulic et al., 2016; Lamas et al., 2019). Recent studies have shown that thermosensitive transient receptor potential channels (ThermoTRPs) play an important role in this process (Song et al., 2016). ThermoTRPs are involved in converting temperature signals to electrical signals and are considered to be a cell “sensor” (Wang and Siemens, 2015; Song et al., 2016; Castillo et al., 2018). There are four representative ThermoTRPs: TRPA1 with nociceptive cold stimulation (<18°C), TRPM8 with non-traumatic cold stimulation (23–28°C), TRPM2 with non-nociceptive thermal stimulation (35–42°C), and TRPV1 with nociceptive thermal stimulation (>43°C) (Caterina et al., 1997; Peier et al., 2002; Bandell et al., 2004; del Camino et al., 2010; Wang and Siemens, 2015; Song et al., 2016; Kashio, 2021). Temperature fluctuations during hot flashes occur in a non-harmful range, which suggests that TRPM2 and TRPM8, rather than TRPV1 and TRPA1, are involved in the occurrence of hot flashes. Recent studies have shown that TRPM2 is a widely distributed temperature sensor in the POA (Song et al., 2016; Yang et al., 2021). The addition of a TRPM2 agonist to POA neurons *in vitro* decreased the thermal activation threshold of the neurons, while the addition of a TRPM2 agonist in POA knockout mice decreased their sensitivity to thermal stimulation, increasing the thermal activation threshold of the neurons (Voets, 2016). Studies have shown that TRPM8 agonists and antagonists increase and decrease the body temperature of experimental animals, respectively (Bautista et al., 2007; Dhaka et al., 2007; Tajino et al., 2011; Gavva et al., 2012), with TRPM8-deleted nerve fibers showing significantly reduced cold sensitivity and TRPM8 knockout mice showing a significant decrease in the ability to prevent hypothermia (Bautista et al., 2007). Thus, changes in TRPM2 and TRPM8 may affect the temperature sensitivity of neurons. Our previous research showed that TRPM2 and TRPM8 expression in the POA decreased when estrogen levels were low, and that abnormal TRPM2 function impaired the ability of neurons to initiate heat dissipation (Yang et al., 2021).

Other studies have suggested that the use of estrogen blockers to reduce estrogen levels in the body can induce extracellular calcium influx, leading to an increase in ThermoTRP activity (Yazǧan and Naziroglu, 2017). Thus, the expression and function of ThermoTRPs may be affected by estrogen levels. The most important thermosensitive neurons in the POA are glutamatergic and GABAergic neurons. Therefore, low estrogen levels are likely to affect the number and function of TRPM2 and TRPM8 in glutamatergic and GABAergic neurons, resulting in an imbalance in the ability of these two types of neurons to regulate heat dissipation and production, resulting in hot flashes. However, there have been no reports on the mechanism by which estrogen regulates the expression and function of TRPM2 and TRPM8.

In summary, we hypothesize that during menopause, the number and function of glutamatergic and GABAergic neurons in the hypothalamic POA may undergo abnormal changes, resulting in an inability of the thermoregulatory center to control heat dissipation and production, triggering hot flashes. The above changes are likely associated with estrogen’s regulation of GAD and ThermoTRPs via nuclear receptors.

## Materials and methods

### Animals and drugs

Two hundred eighty female adult (8–10 weeks) Sprague–Dawley rats were acquired from Peking University Health Science Center’s Animal Laboratory. All animals had unrestricted access to drinking water and soy-free food, which was designed to eliminate the influence of phytoestrogens, and were housed at a constant temperature (23 ± 2°C) and constant humidity (45–55%) under a 12-h light/dark cycle. All research procedures conformed to the National Institutes of Health Guide for the Care and Use of Laboratory Animals and were authorized by the Peking University Biomedical Ethics Committee for Animal Use and Protection.

All rats were kept in the laboratory and allowed to acclimatize for three days before surgery. The 240 rats were divided into three groups: sham surgery with a vehicle (SHAM, *n* = 82), ovariectomy with a vehicle (OVX, *n* = 79), and ovariectomy with estrogen (OVX + E, *n* = 79). The sham animals underwent a bilateral laparotomy but their ovaries were not removed, while the animals in the other two groups underwent a bilateral ovariectomy with a ventral approach. Both the sham surgeries and the ovariectomies were conducted with an intraperitoneal injection of 1% sodium pentobarbital at a dose of 80 mg/kg under strict sterile procedures (Wang et al., 2015; Zhang et al., 2017). To verify the success of the ovariectomy, vaginal exfoliated cells were monitored for seven consecutive days using hematoxylin staining starting on the 3rd day after the ovariectomy to ensure that the ovariectomized rats had no estrous cycle, that estrogen levels were reduced and that the ovaries were completely removed (Wang et al., 2015; Zhang et al., 2017). Surgeries were performed as previously described (Sun et al., 2020).

Following a two-week postoperative recovery period, all rats received four weeks of subcutaneous injections. The OVX + E group received β-estradiol (Sigma, E8875) at a dose of 25 μg/kg/day, with β-estradiol dissolved in sterile sesame oil (Acros, 241002500), while the SHAM and OVX groups received an equivalent volume of sesame oil (Sun et al., 2020). The drug dosage was adjusted in accordance with the body weight of the animal and injected between 8:30 and 9:30 am.

### Measurements of serum 17β-estradiol concentration

Two weeks after the ovariectomy, 30 rats (*n* = 10 per group) were randomly selected and anesthetized according to the abovementioned method. Then, blood (500 μl) was collected through the internal canthus vein, incubated at 37°C for 30 min, and centrifuged at 3000 rpm for 15 min at 4°C. The serum 17β-estradiol concentration was measured in the supernatant using commercially available radioimmunoassay kits (Victor) according to the manufacturer’s instructions. Four weeks after treatment, the serum 17β-estradiol concentration was measured again in same 30 rats.

### Measurements of uterine wet weight

After four weeks of treatment, the 30 rats (*n* = 10 per group) used to determine the serum 17β-estradiol concentration were anesthetized according to the above method and decapitated. The uterus (including the vagina) was separated via layer-by-layer laparotomy; then, the surrounding connective tissue was removed, and the absolute wet weight of the uterus was recorded. To account for the effect of the rat body weight on the uterine wet weight, the uterine wet weight coefficient (uterine wet weight coefficient = uterine wet weight/self-weight) was used to compare changes in the uterine wet weights among the three groups (Li et al., 2020).

### Screening and bioinformatics analysis of differentially expressed proteins in the hypothalamus

After four weeks of treatment, 27 rats (*n* = 9 per group) were randomly anesthetized according to the above method and decapitated. The hypothalamus was immediately removed from the brain, and 3 samples were mixed with a liquid nitrogen grinder. The protein extraction and digestion, TMT labeling of the peptides, high pH reversed-phase fractionation and LC–MS/MS analysis were performed as previously described by our team (Jiang et al., 2018). The information for the functional enrichment analysis of the differentially expressed proteins (DEPs) was obtained from the Kyoto Encyclopedia of Genes and Genomes (KEGG). The protein–protein interaction (PPI) networks were constructed using the STRING database and the Cytoscape software program.

### RNAscope detection assay

Glutamatergic neurons were labeled with vesicular glutamate transporter 2 (Vglut2) probes, GABAergic neurons were labeled with vesicular GABA transporter (Vgat) probes, and nuclei were stained with DAPI (Freedman and Dinsay, 2000; Winter and Ueda, 2008). Four weeks after the treatment, 15 rats (n = 5 per group) were randomly selected, anesthetized, and cardially perfused with normal saline (0.9%, 4°C) and paraformaldehyde (4%, 4°C) to access the brain. Brains were postfixed in 4% polyformaldehyde for 8–12 h. The fixed tissue blocks were then placed in a 30% sucrose solution at 4°C until they sank to the bottom. A freezing microtome (Leica, 1900, Wetzlar, Germany) was used to obtain 10-μm-thick slices from the optimal cutting temperature (OCT)-embedded blocks. The four best POA slices from each rat were kept; two were randomly selected to incubated with a mixture of Vglut2 probes (channel 1, C1), TRPM8 probes (channel 2, C2), and TRPM2 probes (channel 3, C3), while the remaining two were incubated with a mixture of Vgat probes (C1), TRPM8 probes (C2), and TRPM2 probes (C3). RNAscope was performed as previously described (Yang et al., 2021).

A confocal microscope (TCS SP5 II, Leica, Wetzlar, Germany) was used for imaging, and two fields of view were randomly selected from each section. DAPI-labeled nuclei (blue) were used as a reference in the statistical analyses. Thus, depending on the probe, a green signal perinucleus identified glutamatergic or GABAergic neurons, a red signal perinucleus identified TRPM2 mRNA expression, and a cyan signal perinucleus identified TRPM8 mRNA expression. An overlap in the green and red signals indicated TRPM2 expression in glutamatergic or GABAergic neurons. An overlap in the green and cyan signals indicated TRPM8 expression in glutamatergic or GABAergic neurons.

### Immunofluorescence staining

Four weeks after treatment, 15 rats (*n* = 5 per group) were randomly selected, and the four best POA slices (20 μm) were collected in the same manner as described for RNAscope. All sections were placed in 0.3% Triton at 37°C for 40 min. After washing in PBS, two slices were randomly selected from each group, incubated in donkey serum at room temperature for 1 h, and then incubated with GAD1 primary antibody overnight at 4°C. The remaining two slices were incubated in goat serum and incubated with rabbit anti-GAD2 primary antibody. After washing, the sections were incubated in corresponding fluorescent secondary antibodies at room temperature for 3 h, and the slices were mounted as described above. Images were observed under a fluorescence confocal microscope, and two fields of view were used for each brain slice. Details on the primary and secondary antibodies are shown in Supplementary Table 1.1.

### Combination of RNAscope and immunofluorescence staining

Four weeks after treatment, 10 rats were randomly selected from SHAM group, and the four best POA slices (10 μm) were collected in the same manner as described above for RNAscope. First, slices from five rats were randomly selected from SHAM group and incubated with anti-ERα primary antibody according to immunofluorescence. After washing, two slices from each rat were randomly selected and incubated with the Vglut2 probe (C1) and stained with Opal 620 s (green) according to RNAscope, while the other two slices were incubated with Vgat probe (C1) and stained with Opal 620 s (green). The sections of the remaining five rats in each group were first incubated with anti-ERβ primary antibody according to immunofluorescence, then incubated with Vglut2 and Vgat probes and stained with Opal 620 (green). Next, we incubated the corresponding fluorescent secondary antibodies against ERα (594, red) and ERβ according to immunofluorescence. These slices were mounted as described above. DAPI-labeled nuclei (blue) were used as a reference in the statistical analyses. Thus, a green signal perinucleus identified glutamatergic or GABAergic neurons, depending on the probe, and a red signal perinucleus identified ERα or ERβ protein expression, depending on the primary antibody. An overlap in the green and red signals indicated ERα or ERβ expression in glutamatergic or GABAergic neurons. Details on the primary and secondary antibodies are shown in Supplementary Table 1.1.

### Western blot assays

Four weeks after treatment, 15 rats (*n* = 5 per group) were randomly selected, anesthetized and decapitated. The brain was immediately removed, and coronal sections of the POA region were punched using a rat brain metal mold according to coordinates from the rat brain atlas. The following steps, including protein extraction, determination of protein concentration, gel electrophoresis, membrane transfer, incubation with primary and secondary antibodies and acquisition of chemiluminescent signals, were carried out as previously described (Sun et al., 2020). The antibodies included primary antibodies against Vglut2, Vgat, GAD1, GAD2, ERα, ERβ, GPR30, and β-actin and their corresponding secondary antibodies. Details on the primary and secondary antibodies are shown in Supplementary Table 1.1. After the absolute gray value of each band was obtained, the relative gray value was determined by dividing the absolute gray value of the target protein by the absolute gray value of β-actin.

### RNA extraction and qRT-PCR

Four weeks after treatment, 15 rats (*n* = 5 per group) were randomly selected, and their POA tissue was obtained with the same method as described above. The total RNA was isolated from the POA tissue using a TransZol UP Plus RNA Kit. The cDNA was synthesized with an EasyScript^®^ All-in-One First Strand cDNA Synthesis SuperMix for qPCR Kit, and qRT–PCR was performed using a PerfectStart™ Green Supermix Kit on a CFX96 Touch Real-Time PCR (Bio–Rad Laboratories, Inc., California, USA). The samples were denatured at 94°C for 30 s, annealed at 94°C for 5 s, and extended at 60°C for 30 s for a total of 45 cycles. The samples were quantified using Bio-Rad CFX Manager (Bio-Rad Laboratories, Inc., CA, USA), with GAPDH as a normalization control. All kits were purchased from TransGen Biotech (Beijing) Co., Ltd. The details on the primers of *vglut2*, *vgat*, *gad1* and *gad2* are shown in Supplementary Table 1.2.

### Stereotactic injection of viral constructs into the preoptic area

A chemogenetic designer receptor exclusively activated by designer drugs (DREADD) receptor-assisted approach was chosen, which allowed us to activate glutamatergic and GABAergic neurons remotely. One week after treatment, 90 rats (*n* = 30 per group) were randomly selected and equally divided into a Vglut2-control virus injection (GLU-Go) group, a Vgat-control virus injection (GABA-Go) group, a Vglut2-activation virus injection (GLU-Gq) group, a Vglut2-inhibition virus injection (GLU-Gi) group, a Vgat-activation virus injection (GABA-Gq) group and a Vgat-inhibition virus injection (GABA-Gi) group. Surgical procedures were performed aseptically, and the rats were anesthetized as described above. The fur on the head of the rats was removed using depilatory cream. The head of the rat was fixed with a brain stereotaxic apparatus (Kopf Model 1900, Tujunga, CA, USA), and the standard midline and zero plane were determined. The rat brain stereotaxic atlas was used to determine the location of the POA. After a minimal area of the skull was exposed, a 0.2 mm-diameter hole was drilled on each side of the POA using a frame-mounted drill (Kopf 1911; Tujunga, CA, USA). Both sides of the POA were injected with viruses at a rate of 100 nl/min for 5 min, thus injecting a total of 500 nl per side. The needle was withdrawn 0.1 mm every 10 s after it was held in place for 10 min. After the viral injection, the scalp was carefully stitched with sterile absorbable-needled sutures. During the operation, the rats were placed on a 37°C heating pad. The specific sequences of the viruses are shown in Supplementary Table 1.3. All viruses were purchased from BrainVTA (Wuhan) Co., Ltd. The POA of the rat after virus injection is shown in Supplementary Figure 1.

### Monitoring of the skin temperature on the back and tail of the mice

Three weeks after virus injection, rats in the GLU-Go, GABA-Go, GLU-Gq, GLU-Gi, GABA-Gq and GABA-Gi groups were placed in a square open box (30 cm × 30 cm × 30 cm) and allowed to move freely, and their skin temperatures were continuously monitored for 30 min. An infrared thermography camera (FORTRIC 220S; FOTRIC Thermal Imaging Technology Company, Shanghai, China) was placed 30 cm above the open box, which was maintained at a temperature of 22–24°C. Then, the rats were intraperitoneally injected with clozapine-N-oxide (CNO) according to their body weight (0.3 mg/kg) to activate the control virus, activation virus, and inhibition virus previously injected into the POA, thereby activating or inhibiting glutamatergic or GABAergic neurons in the POA. Then, the rats were immediately placed in the square open box, and their behavior was continuously monitored for 2 h. The imaging data were collected and imported into an image processing software (FOTRIC AnalyzIR, FOTRIC Thermal Imaging Technology Company, Shanghai, China). The average skin temperatures of the back (BST) and tail (TST) of the rats during the first 30 min were calculated, and the BST and TST of the rats after CNO injection could be calculated at any given time point. Because our preliminary experiment showed that the body temperature response of the rats could not be detected 2 h after CNO injection, we statistically analyzed the average values of the BST and TST at the seven time points after CNO injection: 0, 20, 40, 60, 80, 100 and 120 min.

### CUT&Tag

Four weeks after treatment, three rats in the SHAM group were randomly selected, and their POA tissue was obtained with the same method as described above. During the experiment, two rats were incubated with mouse anti-ERα primary antibody and mouse anti-ERβ primary antibody, while the other rat served as a control group and was incubated with only the second antibody, not the primary antibody. First, a Nuclear Extraction Kit (SN0020, Solarbio, Beijing, China) was used to extract nuclei from the tissues. The tissue was placed into a homogenizer, and 1.0 ml of precooled lysis buffer and 50 μl of reagent A were added. After grinding, the homogenate was transferred to a centrifuge tube and centrifuged at 700 *g* at 4°C for 5 min. After the supernatant was discarded, 0.5 ml of precooled lysis buffer was added for resuscitation. Then, 0.5 ml of medium buffer was added to a new centrifugal tube, the heavy suspension was absorbed along the pipe wall and carefully added to the centrifugal tube, which was centrifuged at 700 *g* at 4°C for 5 min. After the supernatant was discarded, the nuclear precipitation was obtained by adding 0.5 ml of precooled lysis buffer resuspension precipitation to the nuclear precipitation and centrifuging for 10 min at 1,000 *g*. The precipitation was resuspended in 500 μl of PBS, adding 3.1 μl of 16% formaldehyde to 1% of the solution, then incubated at room temperature for 2 min, adding 15 μl of 2.5 m glycine. After the solution sat at room temperature for 5 min and was centrifuged at 1,300 *g* for 4 min at 4°C, the supernatant was discarded, and the nucleus was suspended in 100 μl of wash buffer. The CUT&Tag Assay Kit used in the experiment was purchased from Vazyme Biotech (Beijing) Co., Ltd. The experimental procedures, including the buffer preparation, ConA bead treatment, nuclear incubation with ConA beads, incubation with primary antibodies (mouse anti-ERα primary antibody, NB300-560, Novus, USA; mouse anti-ERβ primary antibody, NB200-305, Novus, USA), pA/G-Tnp incubation, fragmentation, DNA extraction, library amplification and purification of PCR products, were carried out according to the instructions and previous studies (Bartosovic et al., 2021; Janssens et al., 2021). The obtained library was submitted to Annoroad (Beijing) Co., Ltd., to be sequenced, and the bioinformatics analysis was carried out using the CUT_Tag_tool on the Vazymeyun platform.

### Stereotactic injection of agonists and antagonists of ERα and ERβ into the preoptic area

Four weeks after treatment, 60 rats (*n* = 20 per group) were randomly selected and equally divided into ERα group and ERβ group. Five rats were randomly selected from the SHAM group and OVX + E group of ERα group, the antagonists of ERα (AZD9496, HY-12870, MedChemExpress) were injected into POA, and the agonists of ERα (Propyl pyrazole triol, HY-100689, MedChemExpress) were injected into POA of the five rats randomly selected from the OVX group. Five rats were randomly selected from SHAM group and OVX + E group of ERβ group to inject antagonists of ERβ (PHTPP, HY-103456, MedChemExpress) into POA, and five rats from OVX group were randomly selected to inject agonists of ERβ (Liquiritigenin, HY-N0377, MedChemExpress) into POA. Other rats were given the same volume of cosolvent (10% DMSO + 40% PEG300 + 5% Tween-80 + 45% saline, MedChemExpress) in POA. After 72 h of drug injection, the POA was removed according to the above method, and the mRNA and protein expression of Vglut2 and Vgat in POA were detected by Western blot and qRT-PCR, respectively. The details on the primary and secondary antibodies of vglut2 and vgat are shown in Supplementary Table 1.1, and the details on the primers of vglut2 and vgat are shown in Supplementary Table 1.2.

### Statistical analysis

The data collected from each rat are presented as the mean ± standard deviation (SD). We used SPSS 16.0 software (IBM Corporation, Armonk, NY, USA) for all statistical analyses and GraphPad Prism 7 software (GraphPad Prism Software, La Jolla, CA, USA) for plotting. Kolmogorov–Smirnov tests were used to prove that the data had a normal distribution and an equal variance. Student’s *t*-tests were used to compare the outcomes between two groups, and one-way analysis of variance was used to compare the outcomes among three groups. Pairwise comparisons between the groups were analyzed using least significant difference *post hoc* tests. *P* < 0.05 was considered statistically significant.

## Results

### Criteria for successful ovariectomy

**FIGURE 1:**
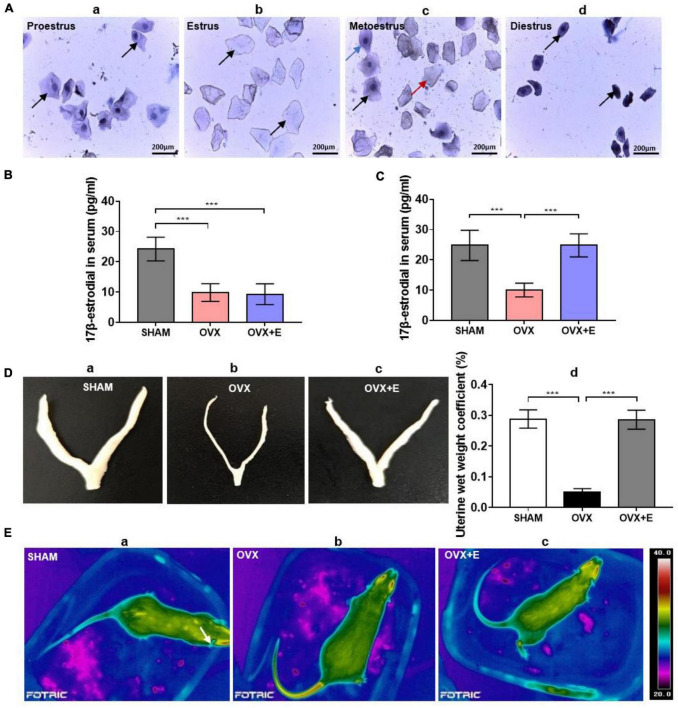
Validation of successful ovariectomies. **(A)** Hematoxylin staining of rat vaginal exfoliated cell smears. (a) Proestrus, the black arrow indicates nucleated epithelial cells; (b) estrus, the black arrow indicates non-nucleated epithelial cells; (c) metoestrus, the black arrow indicates nucleated epithelial cells, the red arrow indicates non-nucleated epithelial cells, and the blue arrow indicates leukocytic cells; (d) diestrous, the black arrow indicates leukocytic cells; scale bar = 200 μm. **(B)** Serum 17β-estradiol concentration of the rats two weeks after ovariectomy; *n* = 10. **(C)** Serum 17β-estradiol concentration of the rats after four weeks of treatment; *n* = 10. **(D)** Uterine morphology and uterine wet weight coefficient of the rats. (a–c) The uterine morphology of rats in SHAM, OVX, and OVX + E groups. (d) Uterine wet weight coefficient of rats; *n* = 10. **(E)** Ovariectomized rats showed symptoms similar to hot flashes. (a) Body skin temperature of rats in the SHAM group. (b) Body skin temperature of rats in the OVX group, with symptoms similar to hot flashes. (c) Body skin temperature of rats in the OVX + E group, with symptoms similar to hot flashes. ANOVA was conducted to compare the outcomes among the three groups, and pairwise comparisons between the groups were conducted using LSD *post hoc* tests. The data are presented as the mean ± SD. *p < 0.05, **p < 0.01, ***p < 0.001. ANOVA, one-way analysis of variance; LSD, least significant difference; OVX, ovariectomy with a vehicle; OVX + E, ovariectomy with estrogen; SD, standard deviation; SHAM, sham surgery with a vehicle.

Two weeks after ovariectomy, hematoxylin staining showed that in the SHAM group, the exfoliated cells in vaginal smears indicated the presence of an estrous cycle, including the proestrus (predominantly nucleated epithelial cells, Figure 1A.a), estrous (predominantly cornified non-nucleated epithelial cells, Figure 1A.b), postestrous (similar proportions of nucleated epithelial cells, cornified non-nucleated epithelial cells, and leukocytic cells, Figure 1A.c), and diestrous (predominantly leukocytic cells, Figure 1A.d) stages. In the OVX and OVX + E groups, the exfoliated cells in vaginal smears always indicated diestrous stage (Figure 1A.d).

Moreover, two weeks after ovariectomy, before the estradiol treatment, the serum 17β-estradiol concentrations of rats in the OVX group and OVX + E group were significantly lower than those of rats in the SHAM group (9.53 ± 3.08 vs 23.24 ± 3.91, *p* < 0.001; 11.13 ± 2.94 vs 23.24 ± 3.91, *p* < 0.001, respectively), and there was no significant difference between the OVX and OVX + E groups (Figure 1B). All analyzed data in this section are shown in Supplementary Table 2.1.

After 4 weeks of estradiol treatment, the serum 17β-estradiol concentration of rats in the OVX group was significantly lower than that of rats in the SHAM group (9.00 ± 1.63 vs. 24.13 ± 4.96, *p* < 0.001). However, the serum 17β-estradiol concentration of rats in the OVX + E group was significantly higher than that of rats in the OVX group (24.00 ± 2.80 vs. 9.00 ± 1.63, *p* < 0.001) but did not differ from that of rats in the SHAM group (Figure 1C). All analyzed data are shown in Supplementary Table 2.2.

The uterine morphology of rats in the OVX group was significantly smaller than that of rats in the SHAM group; however, there was no significant difference between the OVX + E and SHAM groups (Figures 1Da–c). The uterine wet weight coefficient of rats in the OVX group was significantly lower than that of rats in the SHAM group (0.05 ± 0.01% vs. 0.29 ± 0.03%, *p* < 0.001), while that of rats in the OVX + E group was significantly higher than that of rats in the OVX group (0.30 ± 0.03% vs. 0.05 ± 0.01%, *p* < 0.001), and there was no significant difference between the OVX + E and SHAM groups (Figure 1D.d). All analyzed data are shown in Supplementary Table 2.3.

After body temperature was monitored for 24 h, it was found that the TST suddenly increased in the rats of the OVX group, similar to the situation in hot flashes (Figure 1E.b); however, the time and frequency of occurrence were not fixed. There was no similar increase in the SHAM group or OVX + E group (Figure 1E.a,c).

### Kyoto Encyclopedia of Genes and Genomes pathway and interaction network enriched by differentially expressed proteins

**FIGURE 2:**
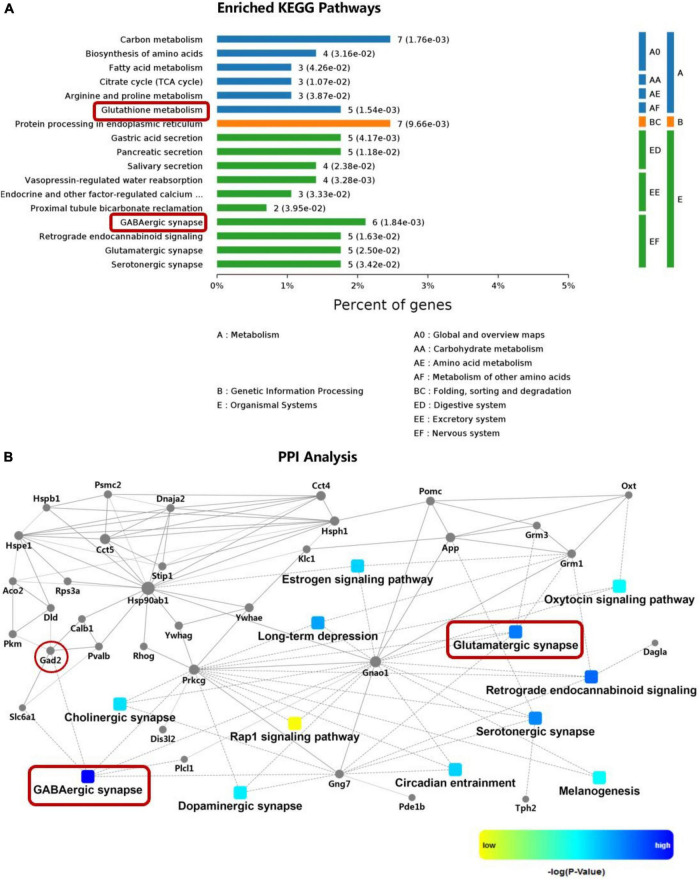
KEGG pathway enrichment and PPI analysis of DEPs in the hypothalamus. **(A)** KEGG pathways enriched for the DEPs. **(B)** PPI analysis of the DEPs. The red boxes indicate glutamatergic synapses and GABAergic synapses, and the red circles indicate glutamate converted into GABA via GAD2. DEPs, differentially expressed proteins; GAD2, glutamate decarboxylase 2; KEGG, Kyoto Encyclopedia of Genes and Genomes; PPI, protein–protein interaction.

A total of 295 DEPs were detected in the hypothalamic tissues of rats in the SHAM, OVX and OVX + E groups (Supplementary Table 2.1); glutamatergic and GABAergic synapses were among the first 17 KEGG pathways and had the largest PPIs corresponding to these DEPs (Figure 2 and Supplementary Tables 2.2, 2.3). GAD2, which converts glutamate to GABA, was also found in the PPI (Figure 2B and Supplementary Table 2.1).

### Number of glutamatergic and GABAergic neurons and Vglut2 and vgat expression in the preoptic area

**FIGURE 3:**
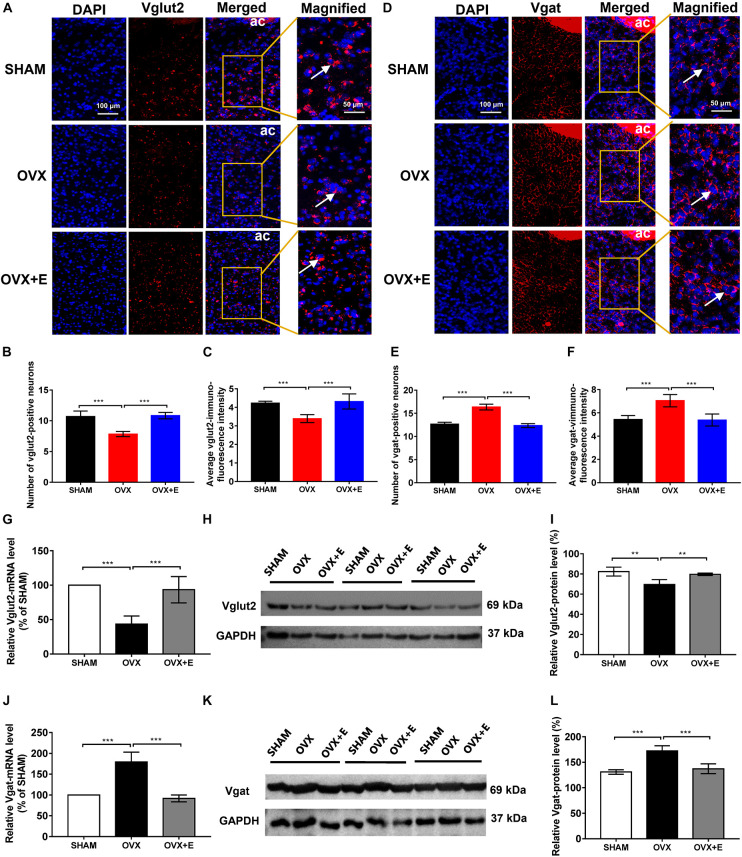
Number of glutamatergic and GABAergic neurons and expression of Vglut2 and Vgat in the POA. **(A)** RNAscope detection of Vglut2. The arrows indicate representative positive neurons; scale bar = 100 or 50 mm. **(B)** Numbers of Vglut2-positive (glutamatergic) neurons in the POA; *n* = 5. **(C)** Average immunofluorescence intensity of Vglut2 in the POA; *n* = 5. **(D)** RNAscope detection of Vgat. The arrows indicate representative positive neurons; scale bar = 100 or 50 mm. **(E)** Numbers of Vgat-positive (GABAergic) neurons in the POA; *n* = 5. **(F)** Average immunofluorescence intensity of Vgat in the POA; *n* = 5. **(G)** Relative mRNA levels of Vglut2 (Vglut2/GAPDH); *n* = 5. **(H)** Immunoblots of Vglut2 (69 kDa) and GAPDH (37 kDa). **(I)** Relative protein levels of Vglut2 (Vglut2/GAPDH); *n* = 5. **(J)** Relative mRNA levels of Vgat (Vgat/GAPDH); *n* = 5. **(K)** Immunoblots of Vgat (69 kDa) and GAPDH (37 kDa). **(L)** Relative protein levels of Vgat (Vgat/GAPDH); *n* = 5. ANOVA was conducted to compare the outcomes among the three groups, and pairwise comparisons between the groups were conducted using LSD *post hoc* tests. The data are presented as the mean ± SD. *p < 0.05, **p < 0.01, ***p < 0.001. Ac, anterior commissure; ANOVA, one-way analysis of variance; LSD, least significant difference; OVX, ovariectomy with a vehicle; OVX + E, ovariectomy with estrogen; POA, preoptic area; SD, standard deviation; SHAM, sham surgery with a vehicle; Vgat, vesicular GABA transporter; Vglut2, vesicular glutamate transporter 2.

The RNAscope results showed that the number of glutamatergic neurons specifically labeled by Vglut2 and the fluorescence intensity of Vglut2 in the POA were significantly lower in the OVX group than in the SHAM group (7.85 ± 0.42 vs. 10.70 ± 0.89, *p* < 0⋅001; 3.39 ± 0.22 vs. 4.23 ± 0.10, *p* < 0.001, respectively), while they were significantly higher in the OVX + E group (10.85 ± 0.52 vs. 7.85 ± 0.42, *p* < 0.001; 4.32 ± 0.41 vs. 3.39 ± 0.22, *p* < 0.001, respectively). There were no differences between the OVX + E and SHAM groups (Figures 3A–C). However, the number of GABAergic neurons specifically labeled by Vgat and the fluorescence intensity of Vgat in the POA were significantly higher in the OVX group than in the SHAM group (16.35 ± 0.63 vs. 12.65 ± 0.42, *p* < 0.001; 7.05 ± 0.53 vs. 5.43 ± 0.34, *p* < 0.001, respectively), while they were significantly lower in the OVX + E group (12.35 ± 0.42 vs. 16.35 ± 0.63, *p* < 0.001; 5.38 ± 0.52 vs. 7.05 ± 0.53, *p* < 0.001, respectively). There were no differences between the OVX + E and SHAM groups (Figures 3D–F).

Similarly, the qRT–PCR and Western blot results showed that Vglut2 expression was significantly lower in the OVX group than in the SHAM group (43.40 ± 11.74 vs. 100, *p* < 0.001; 69.53 ± 4.85 vs. 82.38 ± 4.40, *p* < 0.01, respectively), while Vglut2 expression was significantly higher in the OVX + E group than the OVX group (93.40 ± 19.19 vs. 43.40 ± 11.74, *p* < 0.001; 79.61 ± 1.14 vs. 69.53 ± 4.85, *p* < 0.01, respectively) and did not differ from that in the SHAM group (Figures 3G–I). Furthermore, Vgat expression was significantly higher in the OVX group than in the SHAM group (179.20 ± 23.79 vs. 100, *p* < 0.001; 172.20 ± 10.28 vs. 130.90 ± 4.35, *p* < 0.001, respectively), while Vgat expression in the OVX + E group was significantly lower than in the OVX group (91.80 ± 8.41 vs. 179.20 ± 23.79, *p* < 0.001; 137.20 ± 9.55 vs. 172.20 ± 10.28, *p* < 0.001, respectively) and did not differ from that in the SHAM group (Figures 3J–L).

### Changes in the TST and BST of rats after activating or inhibiting glutamatergic or GABAergic neurons in the preoptic area

Infrared thermal imaging before virus injection showed that the TST and BST values of rats in the OVX group were significantly higher than those in the SHAM group (TST: 31.14 ± 1.10 vs. 30.46 ± 0.03, *p* < 0⋅001; BST: 31.68 ± 0.08 vs. 31.20 ± 0.01, *p* < 0.001), while those in the OVX + E group were significantly lower than those in the OVX group (TST: 30.47 ± 0.09 vs. 31.14 ± 1.10, *p* < 0.001; BST: 31.23 ± 0.06 vs. 31.68 ± 0.08, *p* < 0.001) and did not differ from those in the SHAM group (Supplementary Figure 2). Within 120 min of CNO injection, the TST and BST values of all rats in the GLU-Go and GABA-Go groups did not change significantly (Supplementary Figures 2C–F).

### Changes in the BST and TST of rats after activating or inhibiting glutamatergic neurons in the preoptic area

**FIGURE 4:**
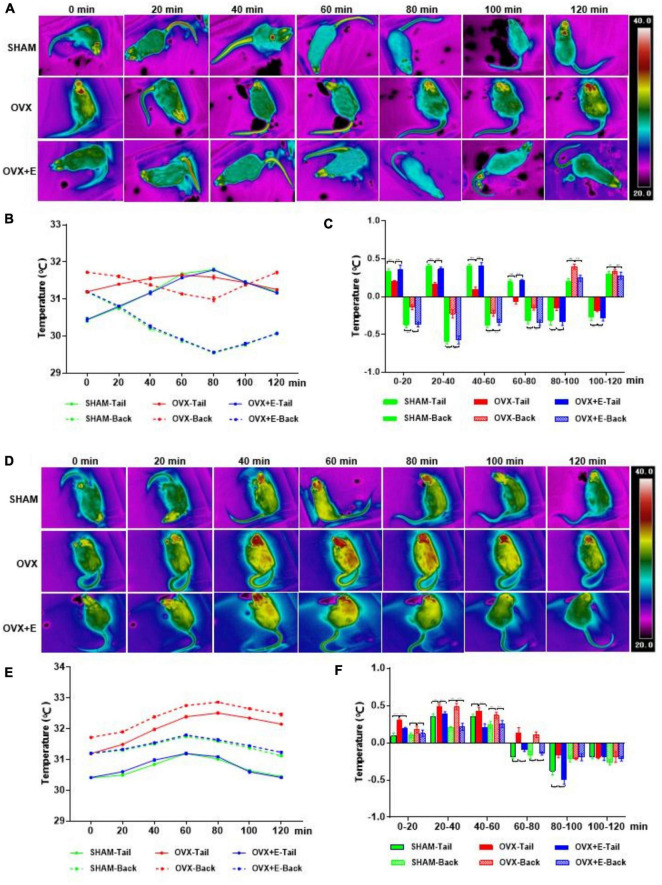
Changes in the TST and BST of rats after activation or inhibition of Glutamatergic neurons in the POA. **(A)** Thermal images of rats in the Vglut2-activation virus injection (GLU-Gq) group every 20 min. **(B,C)** Line chart of the TST and BST and a statistical graph of the range of changes in rats in the GLU-Gq group every 20 min; *n* = 5. **(D)** Thermal images of rats in the Vglut2-inhibition virus injection (GLU-Gi) group every 20 min. **(E,F)** Line chart of the TST and BST and a statistical graph of the range of changes in rats in the GLU-Gi group every 20 min; *n* = 5. ANOVA was conducted to compare the outcomes among the three groups, and pairwise comparisons between the groups were conducted using LSD *post hoc* tests. The data are presented as the mean ± SD. *p < 0.05, **p < 0.01, ***p < 0.001. ANOVA, one-way analysis of variance; BST, skin temperature of the back; LSD, least significant difference; OVX, ovariectomy with a vehicle; OVX + E, ovariectomy with estrogen; POA, preoptic area; SD, standard deviation; SHAM, sham surgery with a vehicle; TST, skin temperature of the tail; Vglut2, vesicular glutamate transporter 2.

After the glutamatergic neurons were specifically activated, the TST increased while the BST decreased in rats in the three groups; then, the TST decreased while the BST increased, and the body temperature gradually returned to normal levels (Figures 4A,B). The TST of the SHAM group increased sharply in the pre-20 min period to dissipate heat. Although the TST decreased gradually from 20 to 80 min, it continued to dissipate heat and then gradually increased; however, the TST did not return to normal levels by 120 min. The BST of the SHAM group continued to decrease during the pre-80 min and then increased gradually; however, the BST did not return to normal levels by 120 min. The trends of the TST and BST in the OVX + E group were similar to those in the SHAM group (Figures 4A–C). After the glutamatergic neurons were specifically inhibited, the TST and BST continuously increased, then decreased gradually and tended toward normal in all three groups (Figures 4D,E). The TST and BST of the SHAM group showed no significant changes in the first 20 min; however, they increased continuously from 20-60 min, then gradually decreased and returned to normal by 120 min. The TST and BST of the OVX group increased during the pre-80 min period, increasing faster than that in the SHAM group (Figures 4D–F); they then gradually decreased but did not return to normal levels by 120 min. The trends of the TST and BST in the OVX + E group were similar to those in the SHAM group.

### Changes in the BST and TST of rats after activating or inhibiting GABAergic neurons in the preoptic area

**FIGURE 5:**
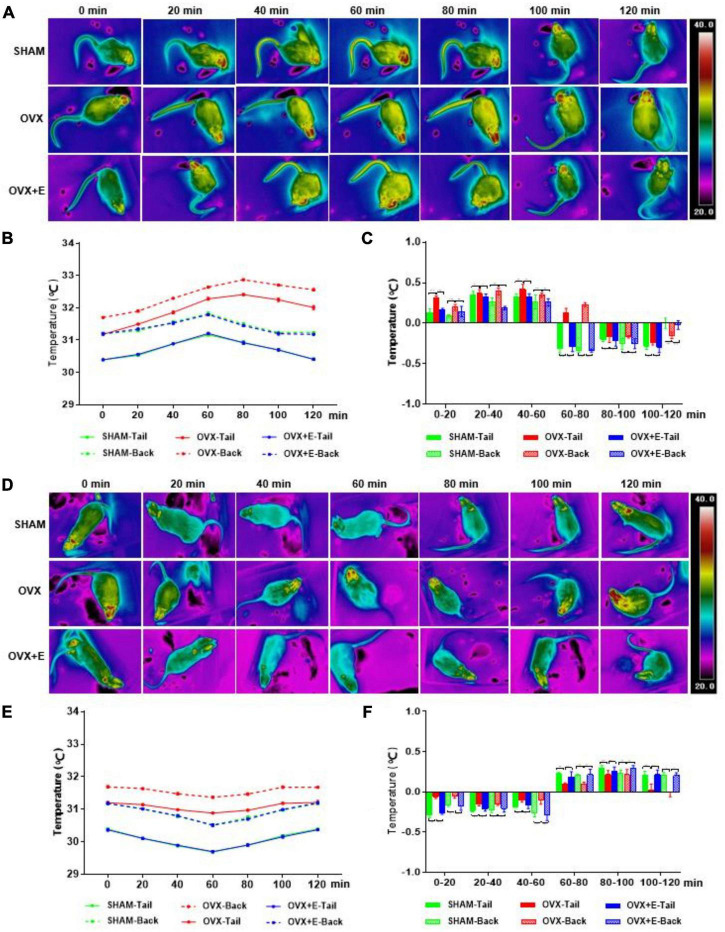
Changes in the TST and BST of rats after activation or inhibition of GABAergic neurons in the POA. **(A)** Thermal images of rats in the Vgat-activation virus injection (GABA-Gq) group every 20 min. **(B,C)** Line chart of the TST and BST and a statistical graph of the range of changes in rats in the GABA-Gq group every 20 min; *n* = 5. **(D)** Thermal images of rats in the Vgat-inhibition virus injection (GABA-Gi) group every 20 min. **(E,F)** Line chart of the TST and BST and a statistical graph of the range of changes in rats in the GABA-Gi group every 20 min; n = 5. ANOVA was conducted to compare the outcomes among the three groups, and pairwise comparisons between the groups were conducted using LSD *post hoc* tests. The data are presented as the mean ± SD. *p < 0.05, **p < 0.01, ***p < 0.001. ANOVA, one-way analysis of variance; BST, skin temperature of the back; LSD, least significant difference; OVX, ovariectomy with a vehicle; OVX + E, ovariectomy with estrogen; POA, preoptic area; SD, standard deviation; SHAM, sham surgery with a vehicle; TST, skin temperature of the tail; Vgat, vesicular GABA transporter.

After the GABAergic neurons were specifically activated, the TST and BST continuously increased and then gradually decreased to normal levels in all three groups (Figures 5A,B). The TST and BST of the SHAM group showed no significant changes during the pre-20 min period; however, they continuously increased from 20 to 60 min, then gradually decreased and returned to normal levels by 100 min. The TST and BST of the OVX group continued to increase during the pre-80 min period, increasing faster than in the SHAM group (Figures 5A–C). The trends of the TST and BST in the OVX + E group were similar to those in the SHAM group. After the GABA neurons were specifically inhibited, the TST and BST continuously decreased and then gradually increased to normal levels (Figures 5D,E). In the SHAM group, the TST and BST decreased slowly during the pre-60 min period, then gradually increased and returned to normal levels by 120 min. The TST and BST in the OVX group showed no obvious changes during the pre-20 min period. However, the TST and BST gradually decreased from 20 to 60 min, decreasing less than in the SHAM group; then, they gradually increased and returned to normal levels by 100 min (Figures 5D–F). The trends of the TST and BST in the OVX + E group were similar to those in the SHAM group.

### GAD1 and GAD2 expression in the preoptic area

**FIGURE 6:**
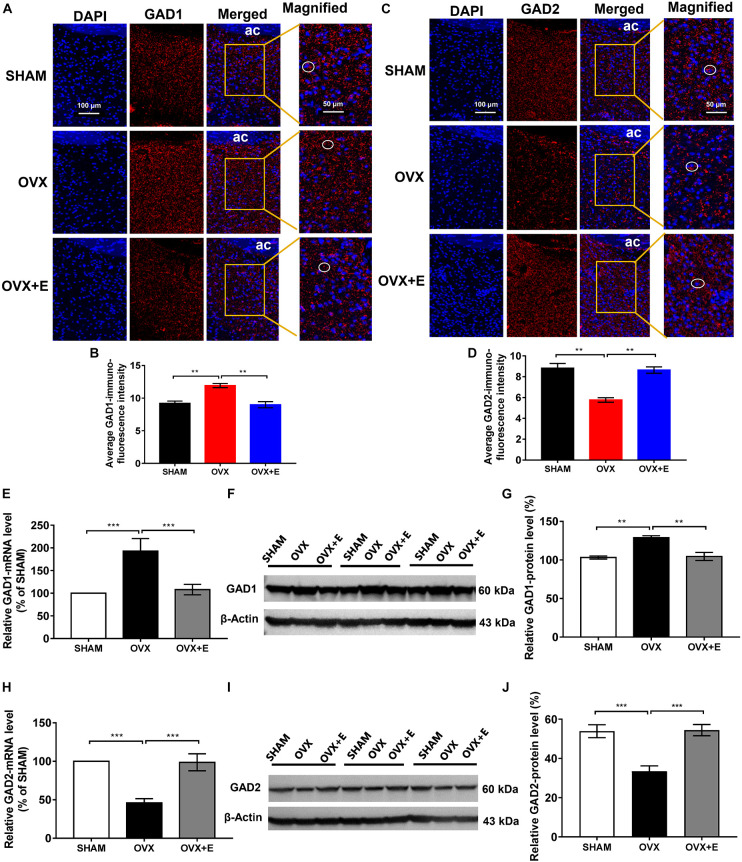
Expression of GAD1 and GAD2 in the POA. **(A)** Immunofluorescence staining of GAD1. The circles indicate representative positive neurons; scale bar = 100 or 50 mm. **(B)** Average immunofluorescence intensity of GAD1 in the POA; *n* = 5. **(C)** Immunofluorescence staining of GAD2. The circles indicate representative positive neurons; scale bar = 100 or 50 mm. **(D)** Average immunofluorescence intensity of GAD2 in the POA; *n* = 5. **(E)** Relative mRNA levels of GAD1 (GAD1/GAPDH); *n* = 5. **(F)** Immunoblots of GAD1 (60 kDa) and β-Actin (43 kDa). **(G)** Relative protein levels of GAD1 (GAD1/β-Actin); *n* = 5. **(H)** Relative mRNA levels of GAD2 (GAD2/GAPDH); *n* = 5. **(I)** Immunoblots of GAD2 (60 kDa) and β-Actin (43 kDa). **(J)** Relative protein levels of GAD2 (GAD2/β-Actin); *n* = 5. ANOVA was conducted to compare the outcomes among the three groups, and pairwise comparisons between the groups were conducted using LSD *post hoc* tests. The data are presented as the mean ± SD. *p < 0.05, **p < 0.01, ***p < 0.001. Ac, anterior commissure; ANOVA, one-way analysis of variance; GAD1, glutamate decarboxylase 2; GAD2, glutamate decarboxylase 2; LSD, least significant difference; OVX, ovariectomy with a vehicle; OVX + E, ovariectomy with estrogen; POA, preoptic area; SD, standard deviation; SHAM, sham surgery with a vehicle.

The immunofluorescence results showed that both GAD1 and GAD2 were located around the nucleus (Figures 6A,C). The fluorescence intensity of GAD1 in the POA was significantly higher in the OVX group than in the SHAM group (11.93 ± 0.32 vs. 9.20 ± 0.34, *p* < 0.01) and was significantly lower in the OVX + E group (8.99 ± 0.48 vs. 11.93 ± 0.32, *p* < 0.01); however, there was no difference between the OVX + E and SHAM groups (Figures 6A,B). The fluorescence intensity of GAD2 in the POA was significantly lower in the OVX group than in the SHAM group (5.77 ± 0.21 vs. 8.82 ± 0.45, *p* < 0.01) and was significantly higher in the OVX + E group (8.64 ± 0.31 vs. 5.77 ± 0.21, *p* < 0.01); however, there was no difference between the OVX + E and SHAM groups (Figures 6C,D).

Similarly, the qRT-PCR and Western blot results showed that GAD1 expression was significantly higher in the OVX group than in the SHAM group (192.96 ± 27.60 vs. 100, *p* < 0.001; 128.85 ± 2.49 vs. 103.13 ± 1.95, *p* < 0.001, respectively), while GAD1 expression was significantly lower in the OVX + E group than in the OVX group (104.40 ± 5.25 vs. 128.85 ± 2.49, *p* < 0.001; 107.97 ± 11.49 vs. 192.96 ± 27.60, *p* < 0.01, respectively) and was not different from that in the SHAM group (Figures 6E–G). Furthermore, GAD2 expression was significantly lower in the OVX group than in the SHAM group (45.95 ± 5.51 vs. 100, *p* < 0.001; 33.46 ± 2.79 vs. 53.88 ± 3.29, *p* < 0.001, respectively), while GAD2 expression in the OVX + E group was significantly higher than that in the OVX group (98.66 ± 11.03 vs. 45.95 ± 5.51, *p* < 0.001; 54.41 ± 2.85 vs. 33.46 ± 2.79, *p* < 0.001, respectively) and was not different from that in the SHAM group (Figures 6H–J).

### Expression of TRPM2 and TRPM8 in glutamatergic or GABAergic neurons in the preoptic area

**FIGURE 7:**
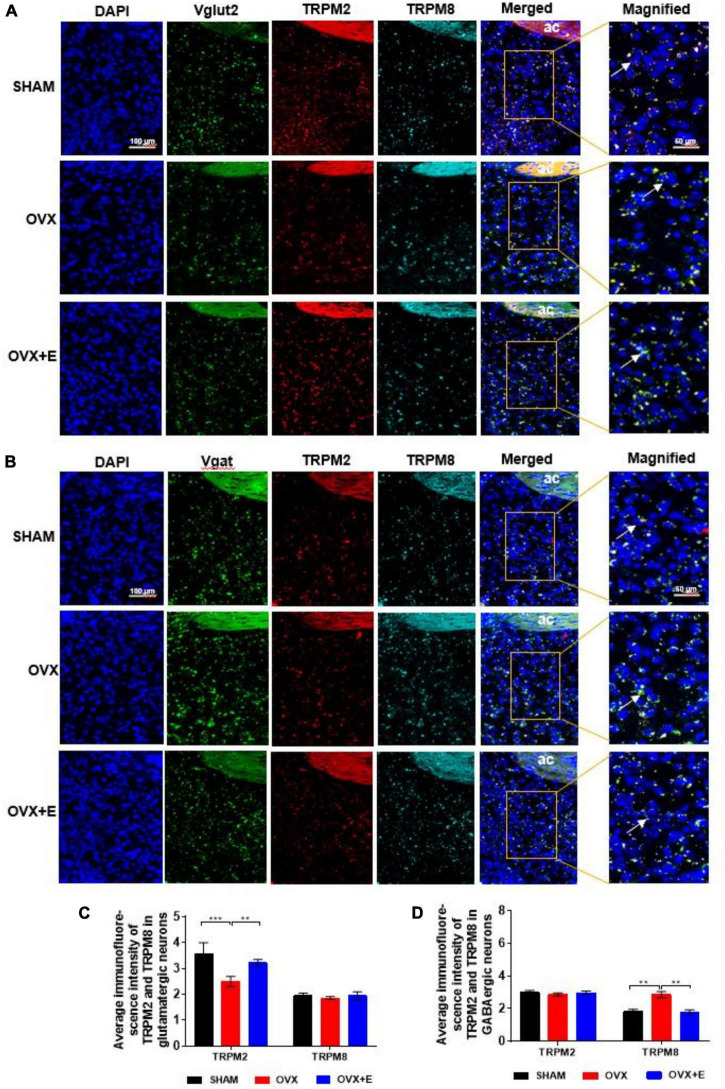
Expression of TRPM2 and TRPM8 in glutamatergic and GABAergic neurons in the POA. **(A,B)** RNAscope detection of Vglut2, TRPM2, and TRPM8 and Vgat, TRPM2, and TRPM8. The arrows indicate representative positive neurons; scale bar = 25 μm. **(C)** Average immunofluorescence intensity of TRPM2 and TRPM8 in glutamatergic neurons; *n* = 5. **(D)** Average immunofluorescence intensity of TRPM2 and TRPM8 in GABAergic neurons; *n* = 5. ANOVA was conducted to compare the outcomes among the three groups, and pairwise comparisons between the groups were conducted using LSD *post hoc* tests. The data are presented as the mean ± SD. *p < 0.05, **p < 0.01, ***p < 0.001. Ac, anterior commissure; OVX, ovariectomy with a vehicle; OVX + E, ovariectomy with estrogen; POA, preoptic area; SHAM, sham surgery with a vehicle; TRPM2, transient receptor potential melastatin-related 2; TRPM8, transient receptor potential melastatin-related 8; Vgat, vesicular GABA transporter; Vglut2, vesicular glutamate transporter 2.

The RNAscope results showed that both TRPM2 and TRPM8 were expressed on glutamatergic and GABAergic neurons. The expression of TRPM2 on glutamatergic neurons was significantly lower in the OVX group than in the SHAM group (2.50 ± 0.19 vs. 3.57 ± 0.44, *p* < 0.001), while the expression in the OVX + E group was significantly higher than that in the OVX group (3.22 ± 0.14 vs. 2.50 ± 0.19, *p* < 0.01) and was not different from that in the SHAM group (Figures 7A,C). However, there was no significant difference in TRPM8 expression on glutamatergic neurons among the three groups (Figures 7A,C). Furthermore, there was no significant difference in TRPM2 expression on GABAergic neurons among the three groups (Figures 7B,D). The expression of TRPM8 on GABAergic neurons was significantly higher in the OVX group than in the SHAM group (2.86 ± 0.19 vs. 1.80 ± 0.15, *p* < 0.01), while that in the OVX + E group was significantly lower than that in the OVX group (1.77 ± 0.14 vs. 2.86 ± 0.19, *p* < 0.01) and was not different from that in the SHAM group (Figures 7B,D).

### Expression of ERα and ERβ on glutamatergic or GABAergic neurons in the preoptic area

**FIGURE 8:**
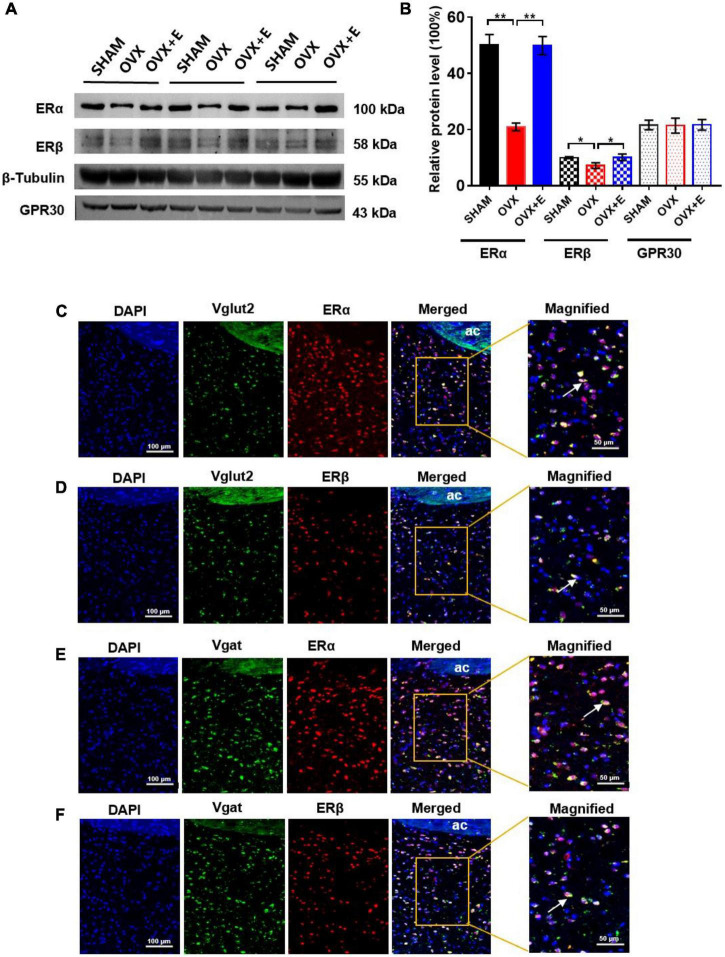
Expression of ERα and ERβ on glutamatergic or GABAergic neurons in the POA. **(A)** Immunoblots of ERα (100 kDa), ERβ (58 kDa), GPR30 (43 kDa), and β-Tubulin (55 kDa). **(B)** Relative protein levels of ERα (ERα/β-Tubulin), ERβ (ERβ/β-Tubulin) and GPR30 (GPR30/β-Tubulin); *n* = 5. **(C)** RNAscope detection of Vglut2 and immunofluorescence staining of ERα. **(D)** RNAscope detection of Vglut2 and immunofluorescence staining of ERβ. **(E)** RNAscope detection of Vgat and immunofluorescence staining of ERα. **(F)** RNAscope detection of Vgat and immunofluorescence staining of ERβ. The arrows indicate representative positive neurons; scale bar = 100 or 50 μm. ANOVA was conducted to compare the outcomes among the three groups, and pairwise comparisons between the groups were conducted using LSD *post hoc* tests. The data are presented as the mean ± SD. *p < 0.05, **p < 0.01, ***p < 0.001. Ac, anterior commissure; ERα, estrogen receptor α; ERβ, estrogen receptor β; OVX, ovariectomy with a vehicle; OVX + E, ovariectomy with estrogen; POA, preoptic area; SHAM, sham surgery with a vehicle.

The Western blot results showed that ERα and ERβ expression was significantly lower in the OVX group than in the SHAM group (20.94 ± 1.40 vs. 50.12 ± 3.73, *p* < 0.01; 7.17 ± 1.03 vs. 10.0 ± 0.35, *p* < 0.05, respectively) in the POA, while ERα and ERβ expression was significantly higher in the OVX + E group than the OVX group (49.87 ± 3.26 vs. 20.94 ± 1.40, *p* < 0.01; 10.08 ± 1.18 vs. 7.17 ± 1.03, *p* < 0.05, respectively) and did not differ from that in the SHAM group (Figures 8A,B). Furthermore, there was no significant difference in the expression of GPR30 in POA among the three groups. The RNAscope and immunofluorescence results showed that both ERα and ERβ were expressed on glutamatergic and GABAergic neurons, and they were both expressed in the nucleus (Figures 8C–F).

### ERα regulated Vglut2 and vgat as a transcription factor

**FIGURE 9:**
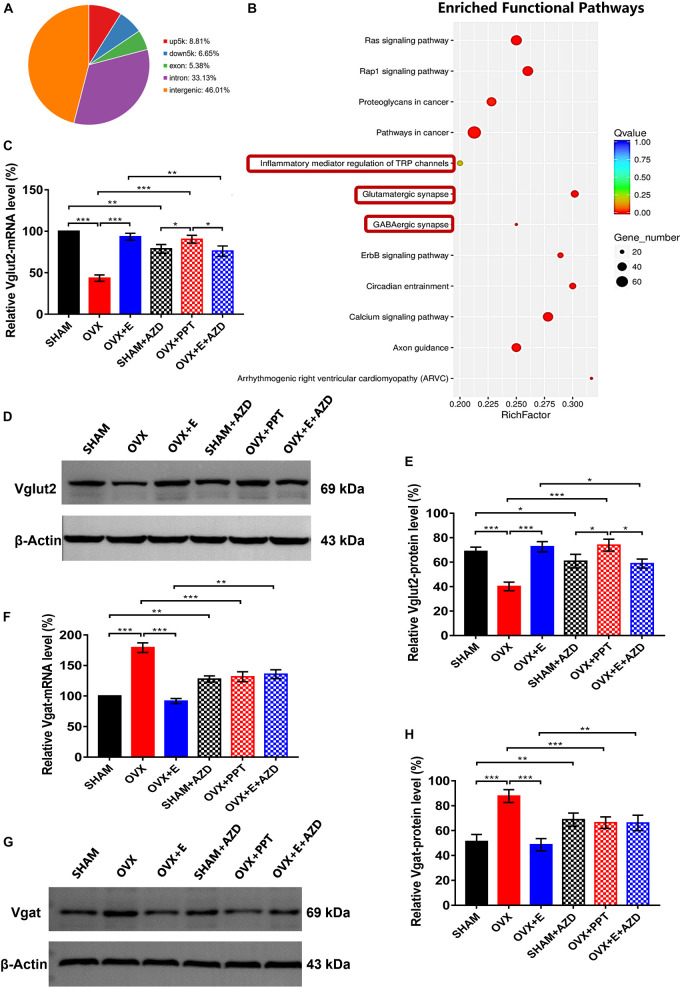
ERα regulated Vglut2 and Vgat as a transcription factor. **(A)** Distribution of peaks pulled down by ERα. **(B)** Pathways enriched by the peaks pulled down by ERα. The red boxes indicate glutamatergic synapses, GABAergic synapses and inflammatory mediators of TRP channels. **(C)** Relative mRNA levels of Vglut2 (Vglut2/β-Actin) before and after the injection of agonists and inhibitors of ERα; *n* = 5. **(D)** Immunoblots of Vglut2 (69 kDa) and β-Actin (43 kDa) before and after the injection of agonists and inhibitors of ERα. **(E)** Relative protein levels of Vglut2 (Vglut2/β-Actin) before and after the injection of agonists and inhibitors of ERα; *n* = 5. **(F)** Relative mRNA levels of Vgat (Vgat/β-Actin) before and after the injection of agonists and inhibitors of ERα; *n* = 5. **(G)** Immunoblots of Vgat (69 kDa) and β-Actin (43 kDa) before and after the injection of agonists and inhibitors of ERα. **(H)** Relative protein levels of Vgat (Vgat/β-Actin) before and after the injection of agonists and inhibitors of ERα; *n* = 5. AZD, AZD9496; ERα, estrogen receptor α; up5k, peaks located within the 5,000 bp upstream of the transcription start site; down5k, peaks located within the 5,000 bp downstream of the transcription start site; PPT, Propyl pyrazole triol; TRP, transient receptor potential.

The CUT&Tag results showed that ERα pulled down 7953 peaks, with 8.81% located within the 5000 bp upstream of the transcription start site (TSS) and 6.65% located within the 5000 bp downstream of the TSS (Supplementary Table 3.1 and Figure 9A). These peaks were enriched in 340 functional pathways, with the top 20 including glutamatergic synapses, GABAergic synapses and TRP channel inflammatory mediators (Supplementary Table 3.2 and Figure 9B).

Before injecting agonists and antagonists of ERα, the qRT–PCR and Western blot results showed that Vglut2 expression was significantly lower in the OVX group than in the SHAM group (43.4 ± 3.87 vs 100, p < 0.001; 40.20 ± 3.46 vs 68.82 ± 3.51, p < 0.05; respectively) in the POA, while Vglut2 expression was significantly higher in the OVX + E group than the OVX group (93.4 ± 4.19 vs 43.4 ± 3.87, p < 0.001; 72.61 ± 4.27 vs 40.20 ± 3.46, p < 0.05; respectively) and did not differ from that in the SHAM group (Figures 9C–E). After injection of ERα antagonist into POA of SHAM and OVX + E groups, the expression of mRNA and protein of Vglut2 decreased significantly (SHAM: 78.8 ± 5.25 vs 100, p < 0.01; 60.82 ± 5.52 vs 68.82 ± 3.51, p < 0.05; respectively; OVX + E: 76.2 ± 6.21 vs 93.4 ± 4.19, p < 0.01; 58.83 ± 3.72 vs 72.61 ± 4.27, p < 0.05; respectively). After injection of ERα agonist into POA of OVX group, the expression of mRNA and protein of Vglut2 increased significantly (90.36 ± 4.69 vs. 43.4 ± 3.87, p < 0.001; 73.92 ± 4.85 vs. 40.20 ± 3.46, p < 0.001; respectively), which was significantly higher than that in SHAM + AZD (*p* < 0.05) and OVX + E + AZD (*p* < 0.05) groups, while there was no significant difference between SHAM + AZD and OVX + E + AZD groups (Figures 9C–E).

Before injecting agonists and antagonists of ERα, Vgat expression was significantly higher in the OVX group than in the SHAM group (179.2 ± 7.94 vs 100, p < 0.001; 87.72 ± 5.15 vs 51.26 ± 5.61, p < 0.001; respectively) in the POA, while Vgat expression was significantly lower in the OVX + E group than the OVX group (91.8 ± 4.08 vs 179.2 ± 7.94, p < 0.001; 48.62 ± 4.92 vs 87.72 ± 5.15, p < 0.001; respectively) and did not differ from that in the SHAM group (Figures 9F–H). After injection of ERα antagonist into POA of SHAM and OVX + E groups, the expression of mRNA and protein of Vgat increased significantly (SHAM: 127.82 ± 5.15 vs 100, p < 0.01; 68.82 ± 5.25 vs 51.26 ± 5.61, p < 0.01; respectively; OVX + E: 135.72 ± 7.32 vs 91.8 ± 4.08, p < 0.01; 66.15 ± 6.21 vs 48.62 ± 5.25, p < 0.01; respectively). After injection of ERα agonist into POA of OVX group, the expression of mRNA and protein of Vgat decreased significantly (131.56 ± 8.14 vs. 179.2 ± 7.94, p < 0.001; 66.36 ± 4.62 vs. 87.72 ± 5.15, p < 0.001; respectively), and there was no significant difference between SHAM + AZD, OVX + PPT and OVX + E + AZD groups (Figures 9F–H).

### ERβ regulated Vglut2 and Vgat as a transcription factor

**FIGURE 10:**
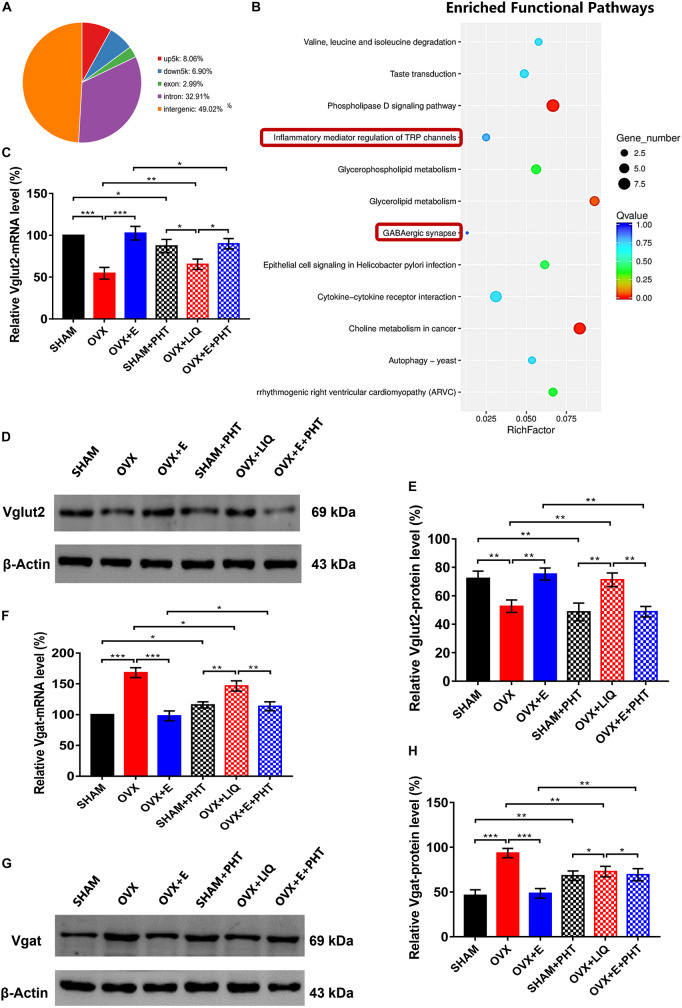
ERβ regulated Vglut2 and Vgat as a transcription factor. **(A)** Distribution of peaks pulled down by ERβ. **(B)** Pathways enriched by the peaks pulled down by ERβ. The red boxes indicate GABAergic synapse and inflammatory mediators of TRP channels. **(C)** Relative mRNA levels of Vglut2 (Vglut2/β-Actin) before and after the injection of agonists and inhibitors of ERβ; *n* = 5. **(D)** Immunoblots of Vglut2 (69 kDa) and β-Actin (43 kDa) before and after the injection of agonists and inhibitors of ERβ. **(E)** Relative protein levels of Vglut2 (Vglut2/β-Actin) before and after the injection of agonists and inhibitors of ERβ; *n* = 5. **(F)** Relative mRNA levels of Vgat (Vgat/β-Actin) before and after the injection of agonists and inhibitors of ERβ; *n* = 5. **(G)** Immunoblots of Vgat (69 kDa) and β-Actin (43 kDa) before and after the injection of agonists and inhibitors of ERβ. **(H)** Relative protein levels of Vgat (Vgat/β-Actin) before and after the injection of agonists and inhibitors of ERβ; *n* = 5. ERβ, estrogen receptor β; up5k, peaks located within the 5,000 bp upstream of the transcription start site; down5k, peaks located within the 5,000 bp downstream of the transcription start site; LIQ, Liquiritigenin; PHT, PHTPP; TRP, transient receptor potential.

The CUT&Tag results showed that ERβ pulled down 868 peaks, with 8.06% located within the 5,000 bp upstream of the TSS and 6.90% located within the 5,000 bp downstream of the TSS (Supplementary Table 4.1 and Figure 10A). These peaks were enriched in 188 functional pathways, with the top 20 pathways including GABAergic synapses and TRP channel inflammatory mediators (Supplementary Table 4.2 and Figure 10B).

Before injecting agonists and antagonists of ERβ, the qRT-PCR and Western blot results showed that Vglut2 expression was significantly lower in the OVX group than in the SHAM group (54.61 ± 6.92 vs. 100, *p* < 0.001; 52.64 ± 4.36 vs. 72.25 ± 5.14, *p* < 0.01, respectively) in the POA, while Vglut2 expression was significantly higher in the OVX + E group than the OVX group (102.52 ± 8.20 vs. 54.61 ± 6.92, *p* < 0.001; 75.26 ± 4.26 vs. 52.64 ± 4.36, *p* < 0.01, respectively) and did not differ from that in the SHAM group (Figures 10C–E). After injection of ERβ antagonist into POA of SHAM and OVX + E groups, the expression of mRNA and protein of Vglut2 decreased significantly (SHAM: 87.14 ± 7.92 vs. 100, *p* < 0.05; 46.68 ± 6.25 vs. 72.25 ± 5.14, *p* < 0.01, respectively; OVX + E: 89.86 ± 6.19 vs. 102.52 ± 8.20, *p* < 0.05; 48.83 ± 3.72 vs. 75.26 ± 4.26, *p* < 0.01, respectively). After injection of ERβ agonist into POA of OVX group, the expression of mRNA and protein of Vglut2 increased significantly (65.26 ± 6.25 vs. 54.61 ± 6.92, *p* < 0.01; 71.25 ± 4.85 vs, 52.64 ± 4.36, *p* < 0.01, respectively), which was significantly higher than that in SHAM + PHT (mRNA: *p* < 0.05; protein: *p* < 0.01) and OVX + E + PHT (mRNA: *p* < 0.05; protein: *p* < 0.01) groups, while there was no significant difference between SHAM + PHT and OVX + E + PHT groups (Figures 10C–E).

Before injecting agonists and antagonists of ERβ, Vgat expression was significantly higher in the OVX group than in the SHAM group (168.26 ± 7.94 vs. 100, *p* < 0.001; 93.50 ± 5.21 vs. 46.25 ± 6.27, *p* < 0.001, respectively) in the POA, while Vgat expression was significantly lower in the OVX + E group than the OVX group (98.12 ± 8.04 vs. 168.26 ± 7.94, *p* < 0.001; 48.53 ± 5.43 vs. 93.5 ± 5.21, *p* < 0.001, respectively) and did not differ from that in the SHAM group (Figures 10F–H). After injection of ERβ antagonist into POA of SHAM and OVX + E groups, the expression of mRNA and protein of Vgat increased significantly (SHAM: 115.82 ± 5.11 vs. 100, *p* < 0.05; 68.22 ± 5.29 vs. 46.25 ± 6.27, *p* < 0.01, respectively; OVX + E: 113.57 ± 7.32 vs. 98.12 ± 8.04, *p* < 0.05; 69.38 ± 6.76 vs. 48.53 ± 5.43, *p* < 0.01, respectively). After injection of ERβ agonist into POA of OVX group, the expression of mRNA and protein of Vgat decreased significantly (146.56 ± 8.14 vs. 168.26 ± 7.94, *p* < 0.05; 72.86 ± 5.81 vs. 93.5 ± 5.21, *p* < 0.01, respectively), which was still significantly higher than that in SHAM + PHT (mRNA: *p* < 0.01; protein: *p* < 0.05) and OVX + E + PHT (mRNA: *p* < 0.01; protein: *p* < 0.05) groups, while there was no significant difference between SHAM + PHT and OVX + E + PHT groups (Figures 10F–H).

## Discussion

The results of this experiment showed that ovariectomized rats had no estrous cycle and always showed the diestrous stage, that they had significantly reduced serum estradiol concentrations and uterine wet weight coefficients, and that estrogen supplementation was effective, confirming the successful establishment of the ovariectomized rat model and the feasibility of subcutaneous estrogen administration. In addition, through continuous monitoring, we found that ovariectomized rats showed sudden, sharp increases in TST and decreases in BST, similar to hot flashes in the face, neck and upper chest region of hot flash patients, who experience sudden heating and decreased body temperature in other parts of the body [50]. However, no similar phenomenon was observed in the estrogen treatment group, which has not been reported in previous studies.

The hypothalamic POA acts as a thermostat to regulate body temperature, with glutamatergic and GABAergic neurons playing key roles (Morrison and Nakamura, 2019). We speculate that when estrogen levels are low, abnormalities in these two types of neurons may lead to dysfunction in the thermoregulatory center, triggering hot flashes. The results showed that DEPs in the hypothalamus were enriched in glutamatergic and GABA synapses as well as the synaptic functional pathways related to glutamate and GABA; moreover, GAD, the key rate-limiting enzyme in the conversion of glutamate to GABA, was found to be involved in both pathways. This provides a reliable basis for speculation on abnormalities in glutamatergic and GABAergic neurons at low estrogen levels. We did not find that ThermoTRPs were closely related to the function of these two types of neurons in DEPs, possibly due to the complexity of the sevenfold transmembrane structure; however, this does not mean that ThermoTRPs do not play a role, necessitating additional experimental verification. Therefore, we conducted an in-depth study to determine whether the number and function of glutamate and GABA neurons in the hypothalamic POA were abnormal, what kind of abnormalities occurred, the mechanism of these abnormalities and whether such abnormalities could cause hot flashes when estrogen levels were low.

Our experiments showed that the number of glutamatergic and GABAergic neurons in the POA of ovariectomized rats significantly decreased and increased, respectively, and that estrogen supplementation significantly corrected this abnormality, suggesting that estrogen can promote glutamatergic neurons and inhibit GABAergic neurons. GAD is known to be a key enzyme in the conversion of glutamate to GABA; thus, increased GAD expression indicates that more glutamate is converted to GABA, with reduced glutamate release and increased GABA release (Ottem et al., 2004). The experimental results showed that GAD1 expression increased in the POA, while GAD2 expression decreased when estrogen levels were low, suggesting that the effects of GAD1 and GAD2 on the conversion of glutamate to GABA may differ. The number of glutamatergic neurons decreased while the number of GABAergic neurons increased in the hypothalamic POA during the hypoestrogenic state; this may be due to the presence of more GAD in the POA, which converts glutamate to GABA.

Therefore, it is possible that increased GAD1 expression may be important in the imbalance of the number of glutamatergic and GABAergic neurons when estrogen levels are low. It has also been suggested that the two isoforms of GAD1 and GAD2 are encoded by separate genes and may differ in terms of expression, localization and activity (Martyniuk et al., 2007). GAD1 focuses on the synthesis of GABA to satisfy metabolic needs and is mainly involved in the reuptake process, while GAD2 mainly regulates the synaptic release of GABA (Soghomonian and Martin, 1998; Yang et al., 2017). The specific mechanisms underlying the two GADs that regulate glutamatergic and GABAergic neurons in the low estrogen state need to be explored in-depth.

Previous studies have confirmed that glutamatergic neurons can be activated by heat signals, thereby inhibiting the thermogenesis process and promoting heat loss, while GABAergic neurons can be activated by cold signals, thereby increasing the thermogenesis process and promoting heat storage (Yang et al., 2020). When we used a viral tool-based chemogenetic approach to specifically activate glutamatergic neurons in the POA, a heat dissipation response was induced in all three groups of rats. Since rats dissipate heat by expanding blood vessels under the skin of the tail to increase blood flow (Gordon, 1990; Oelkrug et al., 2015), the TST increases while the BST decreases when heat dissipation reactions occur. Thus, with CNO failure, the heat dissipation response gradually weakened, and the body temperature gradually returned to normal levels. In contrast, the cooling response of ovariectomized rats was significantly weaker, with a later onset, a slower speed and a smaller amplitude, and body temperatures returned to normal levels sooner. The above results suggest that the ability of glutamatergic neurons to induce heat dissipation is diminished in the low estrogen state. When glutamatergic neurons were specifically inhibited, GABAergic neurons played a more important role. At this time, the heat dissipation process was inhibited in all three groups of rats; the body temperature of all rats increased, with both the TST and BST increasing, inhibiting the heat dissipation process. The warming response gradually weakened, and the body temperature gradually decreased and returned to normal levels. However, the warming response of ovariectomized rats was significantly stronger than that of rats in the sham-operated group due to the inhibition of heat dissipation. The above results indicate that when estrogen levels are low, GABAergic neurons are more likely to initiate thermogenesis. When GABAergic neurons were specifically activated, the rats in all three groups first showed a warming response, and the TST and BST both increased. The warming response gradually weakened, and the body temperature gradually decreased and returned to normal. However, the warming response of the ovariectomized rats was significantly stronger than that of the rats in the sham-operated group. This confirms the enhanced ability of GABAergic neurons to initiate thermogenesis. When GABAergic neurons were specifically inhibited, glutamatergic neurons played a major role. At this time, the thermogenic response was inhibited. The three groups of rats showed a cooling response, and the TST and BST were both reduced. Then, the cooling response gradually weakened, and the body temperature gradually returned to normal. However, the cooling response of the ovariectomized rats was significantly weaker than that of the rats in the sham-operated group due to the inhibition of the thermogenic response. This confirms that the ability of glutamatergic neurons to initiate heat dissipation is diminished in the low estrogen state. In addition, there was no significant difference in responses between the estrogen-supplemented rats and the sham-operated group. In conclusion, when estrogen levels are low, the ability of glutamatergic neurons to initiate heat dissipation in the hypothalamic POA is weakened, while the ability of GABAergic neurons to initiate heat production is enhanced. On the one hand, this may be due to the decrease in the number of glutamatergic neurons and the increase in the number of GABAergic neurons in the POA when estrogen levels are low; on the other hand, they may be due to abnormal TRPM2 and TRPM8 expression in the two types of neurons. The experimental results show that TRPM2 expression in glutamatergic neurons was reduced in the low estrogen state, which likely reduces the ability of glutamatergic neurons to sense thermal stimuli and regulate heat dissipation, while TRPM8 expression in GABAergic neurons was increased, which likely enhances the ability of GABAergic neurons to sense cold stimuli and regulate heat production. The above results suggest that TRPM2 and TRPM8 affect the temperature sensitivity of the neurons in which they reside. Another possibility is that because TRPM8 is a channel that senses cold stimuli (Ordás et al., 2021), its expression in glutamatergic neurons that receive heat signals does not change significantly in low estrogen states; however, because TRPM2 is a channel that senses heat stimuli (Song et al., 2016), its expression in GABAergic neurons that receive cold signals does not change significantly in low estrogen states.

Previous studies have suggested that reductions in estrogen levels can cause hot flashes and that estrogen might act in the hypothalamic POA, the thermoregulatory center of the brain, through its receptors (Freedman, 2000). Furthermore, previous studies have shown that the nuclear receptor of estrogen can mediate the “genotype regulation effect” of estrogen; that is, estrogen diffuses freely through the plasma membrane, binds tightly to the nuclear receptor, and regulates the transcription and expression of target genes by binding to EREs (Krolick et al., 2018). The results of this study showed that when estrogen levels were low, the estrogen nuclear receptors ERα and ERβ decreased in the hypothalamic POA, and they were expressed in both glutamatergic and GABAergic neurons. We also found that ERα functions as a transcription factor in glutamatergic and GABAergic synapses, while ERβ functions as a transcription factor in GABAergic synapses, implying that estrogen regulates glutamatergic and GABAergic neurons through its nuclear receptors. This conclusion was proved again by administration of agonists and antagonists of ERα and ERβ. Under the condition of low estrogen, the expression of ERα and ERβ decreased, the expression of Vglut2 decreased and Vgat increased in POA. After administration of ERα and ERβ agonists, the expression of Vglut2 increased and Vgat

decreased. At the same time, when ERα and ERβ antagonists were given to rats with normal estrogen levels, the expression of Vglut2 decreased and the expression of Vgat increased accordingly. A study using chromatin immunoprecipitation (CHIP) technology found that the promoter region of GAD2 contains ERE (Krolick et al., 2018); however, we did not find that ERα and ERβ could be used as transcription factors for GAD1 and GAD2 in CUT&Tag experiments, which could be due to the different technical principles of CUT&Tag and CHIP. The study have showed that in the ovariectomized rat model treated with estrogen, the mRNA levels of GAD1 and GAD2 in the periventricular nucleus of the forebrain decreased and increased, respectively. At the same time, the expression of glutamatergic neuron specific marker Vglut2 and GABA neuron specific marker Vgat increased and decreased, respectively. These results suggest that estrogen may inhibits the expression of GAD1 and promotes the expression of GAD2, which ultimately reduces the utilization rate of glutamate in the synthesis of GABA, resulting in more glutamate for release. Combined with our results, we can tentatively speculate that estrogen likely regulates the transcription and expression of GAD1 and GAD2 through the nuclear receptors ERα and ERβ, which in turn affects the expression of glutamate and GABA, changing the number of glutamatergic and GABAergic neurons in the hypothalamic POA. However, it is unclear whether the ERE found in the GAD2 promoter region has a function, and whether the GAD1 promoter region contains EREs. Research shows that estrogen can enhance the expression of TRPV1 in endometrial cells through ERα (Ahn et al., 2014), but inhibit the function of TRPV1 in dorsal root ganglion through ERβ (Chakraborty et al., 2017). At the same time, estrogen deficiency can increase the expression of TRPM8 in rat dermis (Kubo et al., 2017). Reduced estrogen levels have been shown to reduce TRPM2 expression in the dorsal root ganglia and hippocampus and to affect the activity and function of TRPM2 (Yazǧan and Naziroglu, 2017). In this study, low estrogen levels reduced TRPM2 expression in glutamatergic neurons in the hypothalamic POA and increased TRPM8 expression in GABAergic neurons, which suggests that estrogen may regulate the expression of ThermoTRPs through a variety of mechanisms. However, ThermoTRPs are membrane channel proteins and the estrogen receptors with expression changes are nuclear receptors, and no research has been done to prove that estrogen nuclear receptors directly participate in the regulation of ThermoTRP expression in the POA; thus, we speculate that there may be a secondary messenger or another signaling pathway between the two. Our results show that both ERα and ERβ regulate inflammatory mediators in TRP channels as transcription factors, suggesting that ERα and ERβ may regulate the expression of TRPM2 and TRPM8 through inflammatory factors; however, the specific molecules and mechanisms need to be investigated further.

## Conclusion

In the classic thermoregulatory pathway, it is true that glutamatergic and GABAergic neurons play a key role in POA, but there are no studies to explore the changes of glutamatergic and GABAergic neurons in the POA under the condition of low estrogen and their relationship with hot flashes. In this study, we first confirmed that glutamatergic neurons initiate heat dissipation and GABA neurons initiate thermogenesis in POA. Secondly, it was found that under the condition of low estrogen, the number and function of glutamatergic neurons in POA decreased, while the number and function of GABA neurons increased, which provided a more accurate target for the mechanism of hot flashes caused by POA dysfunction. Thirdly, it also revealed the molecular mechanism of the participation of ThermoTRPs and GADs in the low estrogen state. In summary, during menopause, a decrease in the estrogen level regulates the increased expression of GAD1 and the decreased expression of GAD2 in the POA via reduced signaling through the nuclear receptors ERα and Erβ, resulting in a decrease in the number of glutamatergic neurons and an increase in the number of GABAergic neurons. Furthermore, the decrease in estrogen may regulate the decreased expression of TRPM2 in glutamatergic neurons and the increased expression of TRPM8 in GABAergic neurons via ERα and ERβ. This leads to functional changes in both types of neurons. Abnormalities in the number and function of glutamatergic and GABAergic neurons lead to imbalances in the hypothalamic POA, the thermoregulatory center of the brain, which regulates heat dissipation and production and ultimately triggers hot flashes. In-depth investigation of this mechanism is expected to reveal the key roles of glutamatergic and GABAergic neurons in the hypothalamic POA during hot flashes as well as the participation mechanisms of GADs and ThermoTRPs, providing a theoretical basis for the screening of new targets for the clinical diagnosis and treatment of menopausal hot flashes.

## Data availability statement

The original contributions presented in the study are included in the article/Supplementary material, further inquiries can be directed to the corresponding authors.

## Ethics statement

The animal study was reviewed and approved by Peking University Biomedical Ethics Committee for Animal Use and Protection.

## Author contributions

LQ, JY, and JJ conceived and supervised this study. LQ was responsible for all aspects of study design. YS conducted the experiments, interpreted all results, and wrote the manuscript. YS and HW performed the statistical analyses. JL, JZ, and XL provided advice. WW, LL, and KW verified the underlying data. All authors critically reviewed the manuscript and agreed to be accountable for the content of the work.
