# Corrigendum: Glutamatergic and GABAergic neurons in the preoptic area of the hypothalamus play key roles in menopausal hot flashes

**DOI:** 10.3389/fnagi.2024.1072608

**Published:** 2024-07-04

**Authors:** Yanrong Sun, Hanfei Wang, Wenjuan Wang, Jiali Lu, Jinglin Zhang, Xiaofeng Luo, Liju Luan, Ke Wang, Jing Jia, Junhao Yan, Lihua Qin

**Affiliations:** ^1^Department of Human Anatomy, Histology and Embryology, School of Basic Medical Sciences, Peking University Health Science Center, Beijing, China; ^2^Department of Stomatology, Shanxi Medical University School and Hospital of Stomatology, Taiyuan, Shanxi, China; ^3^Department of Stomatology, The Third Medical Center, Chinese PLA General Hospital, Beijing, China; ^4^Beijing Key Lab of Magnetic Resonance Imaging Technology, Peking University Third Hospital, Beijing, China

**Keywords:** hot flashes, glutamatergic neurons, GABAergic neurons, glutamate decarboxylase, thermosensitive transient receptor potentials, estrogen receptors

In the published article, there was an error in Figure 3 as published. The wrong slide of microscopy was provided for OVX + E group in Figure 3A. There were incorrect kDa values of Vglut2 and Vgat due to clerical errors, and there were red curves below kDa and Vgat caused by spell checking in Figures 3H, K. The corrected Figure 3 and its caption appear below.

**Figure 3:**
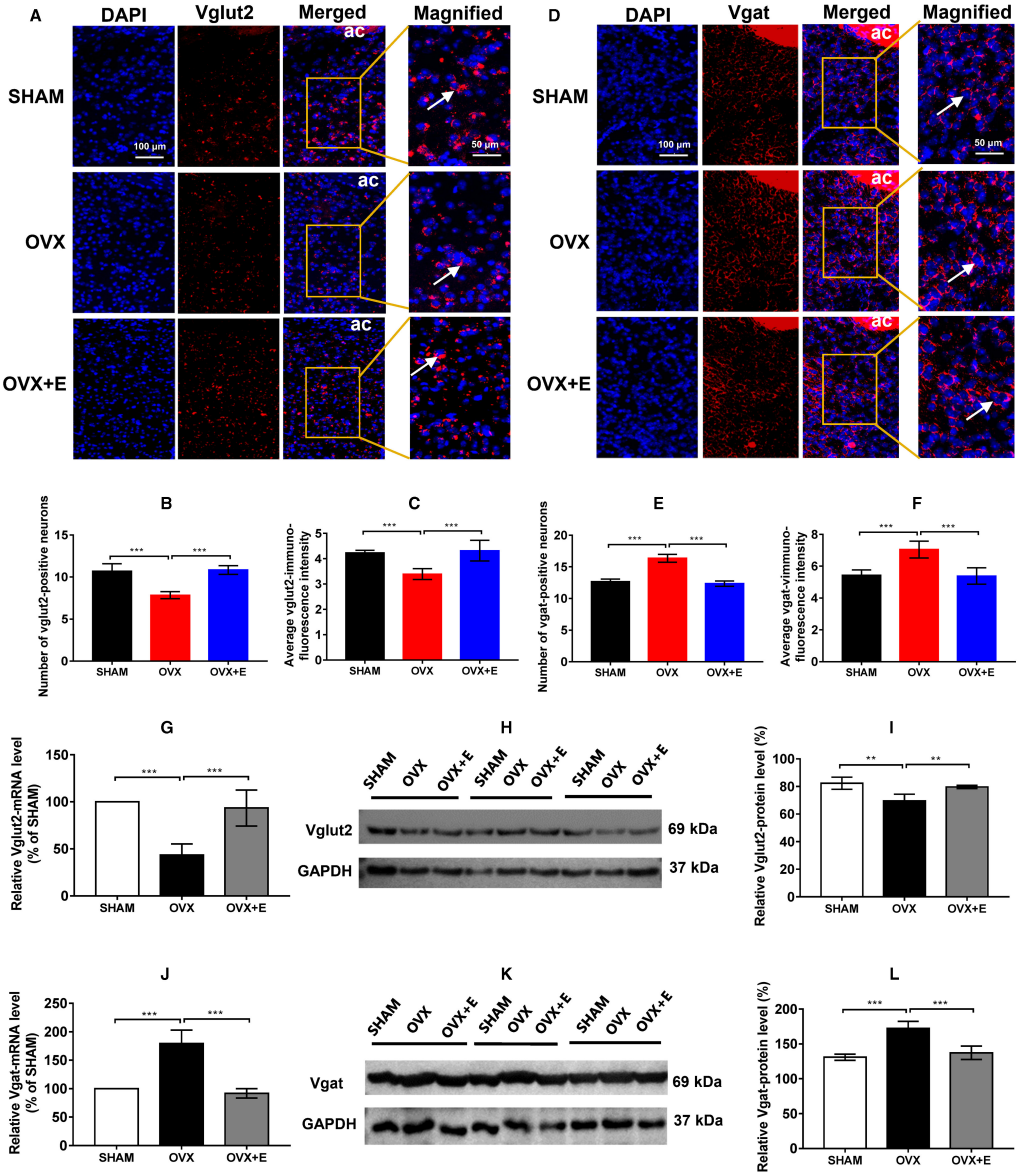
Number of glutamatergic and GABAergic neurons and expression of Vglut2 and Vgat in the POA. Number of glutamatergic and GABAergic neurons and expression of Vglut2 and Vgat in the POA. **(A)** RNAscope detection of Vglut2. The arrows indicate representative positive neurons; scale bar = 100 or 50 mm. **(B)** Numbers of Vglut2-positive (glutamatergic) neurons in the POA; *n* = 5. **(C)** Average immunofluorescence intensity of Vglut2 in the POA; *n* = 5. **(D)** RNAscope detection of Vgat. The arrows indicate representative positive neurons; scale bar = 100 or 50 mm. **(E)** Numbers of Vgat-positive (GABAergic) neurons in the POA; *n* = 5. **(F)** Average immunofluorescence intensity of Vgat in the POA; *n* = 5. **(G)** Relative mRNA levels of Vglut2 (Vglut2/GAPDH); *n* = 5. **(H)** Immunoblots of Vglut2 (69 kDa) and GAPDH (37 kDa). **(I)** Relative protein levels of Vglut2 (Vglut2/GAPDH); *n* = 5. **(J)** Relative mRNA levels of Vgat (Vgat/GAPDH); *n* = 5. **(K)** Immunoblots of Vgat (69 kDa) and GAPDH (37 kDa). **(L)** Relative protein levels of Vgat (Vgat/GAPDH); *n* = 5. ANOVA was conducted to compare the outcomes among the three groups, and pairwise comparisons between the groups were conducted using LSD post hoc tests. The data are presented as the mean ± SD. ^*^p < 0.05, ^**^p < 0.01, ^***^p < 0.001. Ac, anterior commissure; ANOVA, one-way analysis of variance; LSD, least significant difference; OVX, ovariectomy with a vehicle; OVX + E, ovariectomy with estrogen; POA, preoptic area; SD, standard deviation; SHAM, sham surgery with a vehicle; Vgat, vesicular GABA transporter; Vglut2, vesicular glutamate transporter 2.

In the published article, there was an error in Figure 6 as published. There were red curves below kDa caused by spell checking in Figures 6F, I. The corrected Figure 6 and its caption appear below.

**Figure 6:**
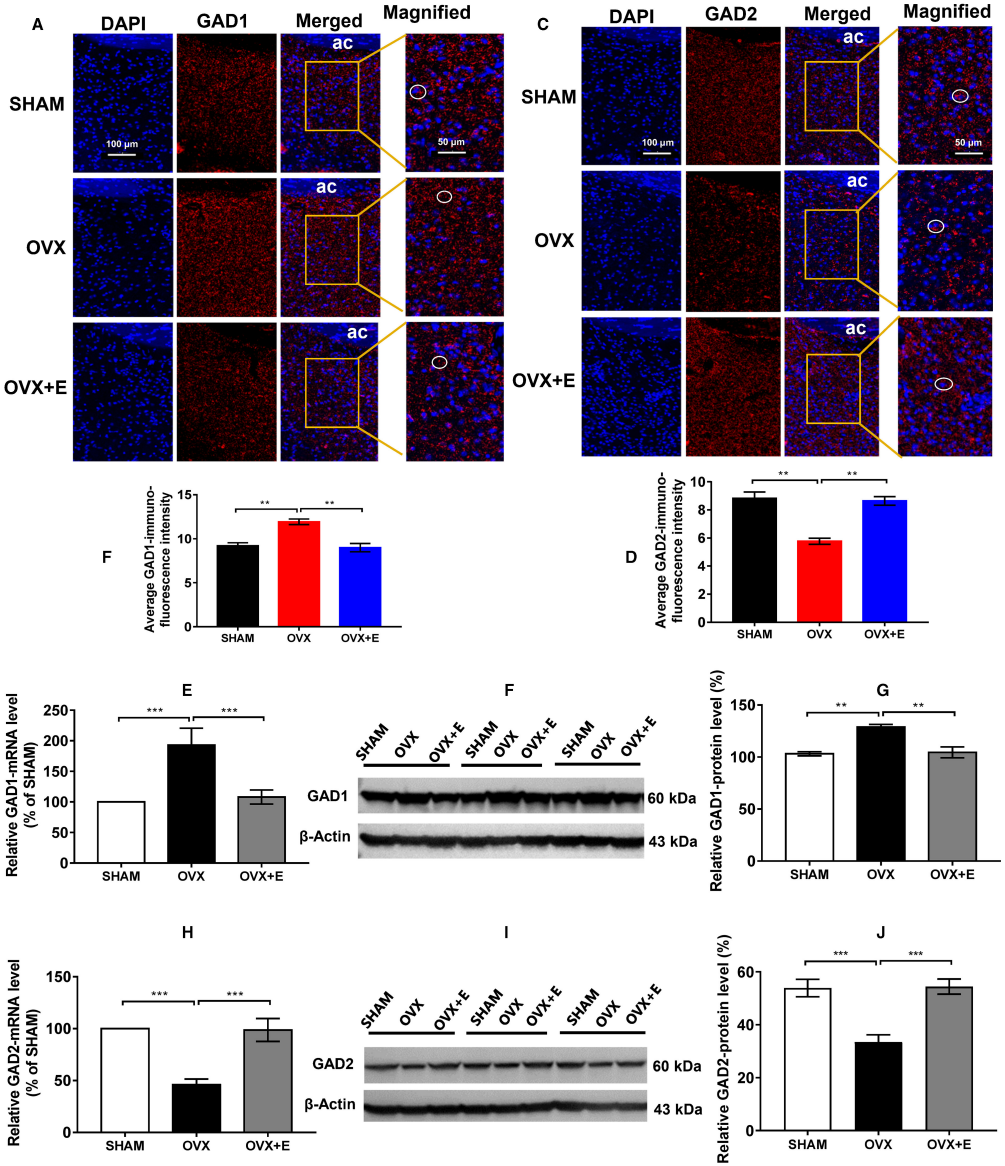
Expression of GAD1 and GAD2 in the POA. **(A)** Immunofluorescence staining of GAD1. The circles indicate representative positive neurons; scale bar = 100 or 50 mm. **(B)** Average immunofluorescence intensity of GAD1 in the POA; *n* = 5. **(C)** Immunofluorescence staining of GAD2. The circles indicate representative positive neurons; scale bar = 100 or 50 mm. **(D)** Average immunofluorescence intensity of GAD2 in the POA; *n* = 5. **(E)** Relative mRNA levels of GAD1 (GAD1/GAPDH); *n* = 5. **(F)** Immunoblots of GAD1 (60 kDa) and β-Actin (43 kDa). **(G)** Relative protein levels of GAD1 (GAD1/β-Actin); *n* = 5. **(H)** Relative mRNA levels of GAD2 (GAD2/GAPDH); *n* = 5. **(I)** Immunoblots of GAD2 (60 kDa) and β-Actin (43 kDa). **(J)** Relative protein levels of GAD2 (GAD2/β-Actin); *n* = 5. ANOVA was conducted to compare the outcomes among the three groups, and pairwise comparisons between the groups were conducted using LSD post hoc tests. The data are presented as the mean ± SD. ^*^p < 0.05, ^**^p < 0.01, ^***^p < 0.001. Ac, anterior commissure; ANOVA, one-way analysis of variance; GAD1, glutamate decarboxylase 2; GAD2, glutamate decarboxylase 2; LSD, least significant difference; OVX, ovariectomy with a vehicle; OVX + E, ovariectomy with estrogen; POA, preoptic area; SD, standard deviation; SHAM, sham surgery with a vehicle.

In the published article, there was an error in Figure 9 as published. There were red curves below kDa and Vgat caused by spell checking in Figures 9D, G, and the abbreviation for ERα antagonist, AZD, was misspelled as ZAD due to an error in Figures 9E, H. And there were incorrect kDa values in the caption due to clerical errors. The corrected Figure 9 and its caption appear below.

**Figure 9:**
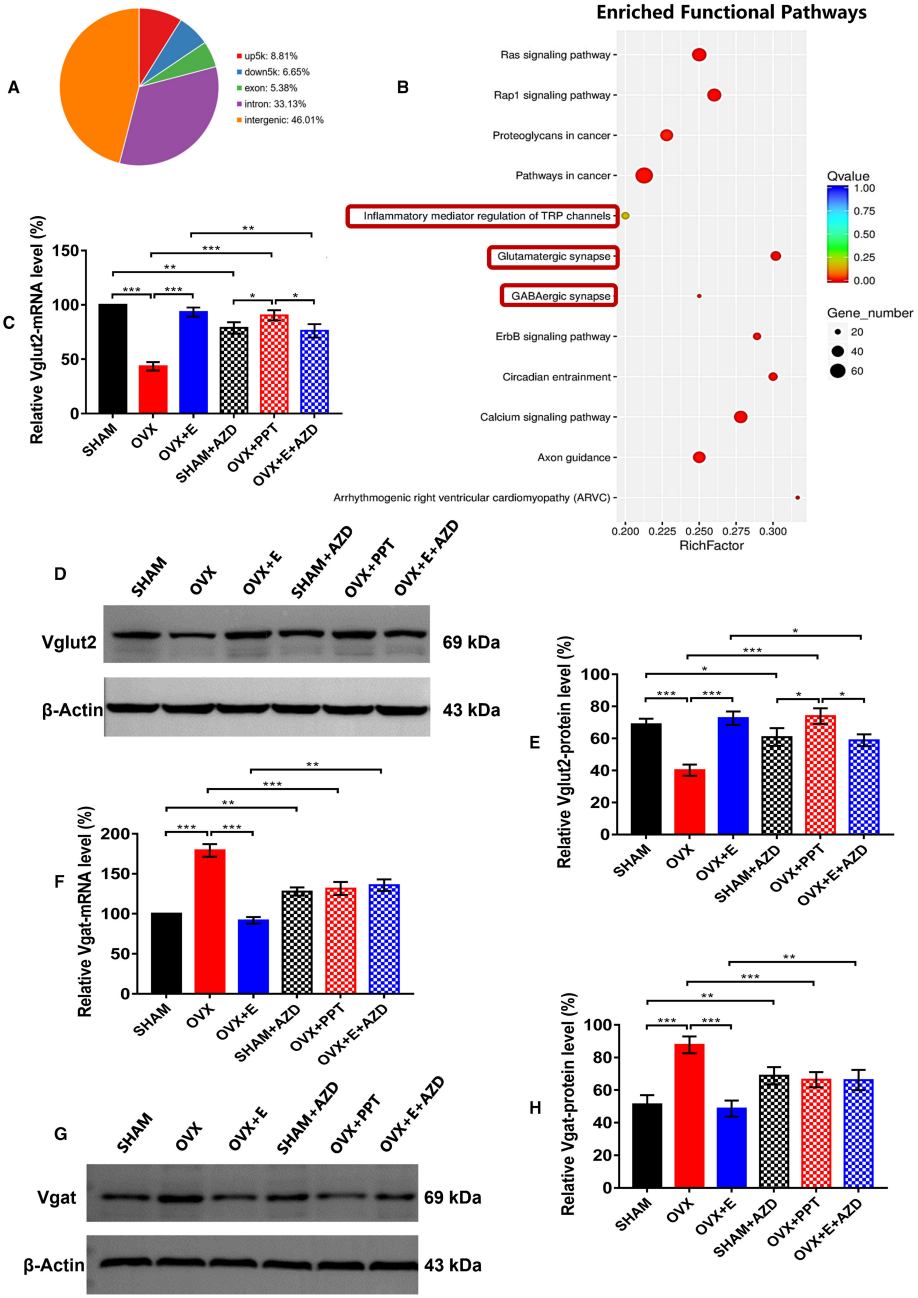
ERα regulated Vglut2 and Vgat as a transcription factor. **(A)** Distribution of peaks pulled down by ERα. **(B)** Pathways enriched by the peaks pulled down by ERα. The red boxes indicate glutamatergic synapses, GABAergic synapses and inflammatory mediators of TRP channels. **(C)** Relative mRNA levels of Vglut2 (Vglut2/b-Actin) before and after the injection of agonists and inhibitors of ERα; *n* = 5. **(D)** Immunoblots of Vglut2 (69 kDa) and β-Actin (43 kDa) before and after the injection of agonists and inhibitors of ERα. **(E)** Relative protein levels of Vglut2 (Vglut2/β-Actin) before and after the injection of agonists and inhibitors of ERα; *n* = 5. **(F)** Relative mRNA levels of Vgat (Vgat/β-Actin) before and after the injection of agonists and inhibitors of ERα; *n* = 5. **(G)** Immunoblots of Vgat (69 kDa) and β-Actin (43 kDa) before and after the injection of agonists and inhibitors of ERα. **(H)** Relative protein levels of Vgat (Vgat/β-Actin) before and after the injection of agonists and inhibitors of ERα; *n* = 5. AZD, AZD9496; ERα, estrogen receptor α; up5k, peaks located within the 5,000 bp upstream of the transcription start site; down5k, peaks located within the 5,000 bp downstream of the transcription start site; PPT, Propyl pyrazole triol; TRP, transient receptor potential.

In the published article, there was an error in Figure 10 as published. There were red curves below kDa and Vgat caused by spell checking in Figures 10D, G, and the abbreviation for ERβ antagonist, PHT, was misspelled as AZD or ZAD due to an error in Figures 10D, G. And there were incorrect kDa values in the caption due to clerical errors. The corrected Figure 10 and its caption appear below.

**Figure 10:**
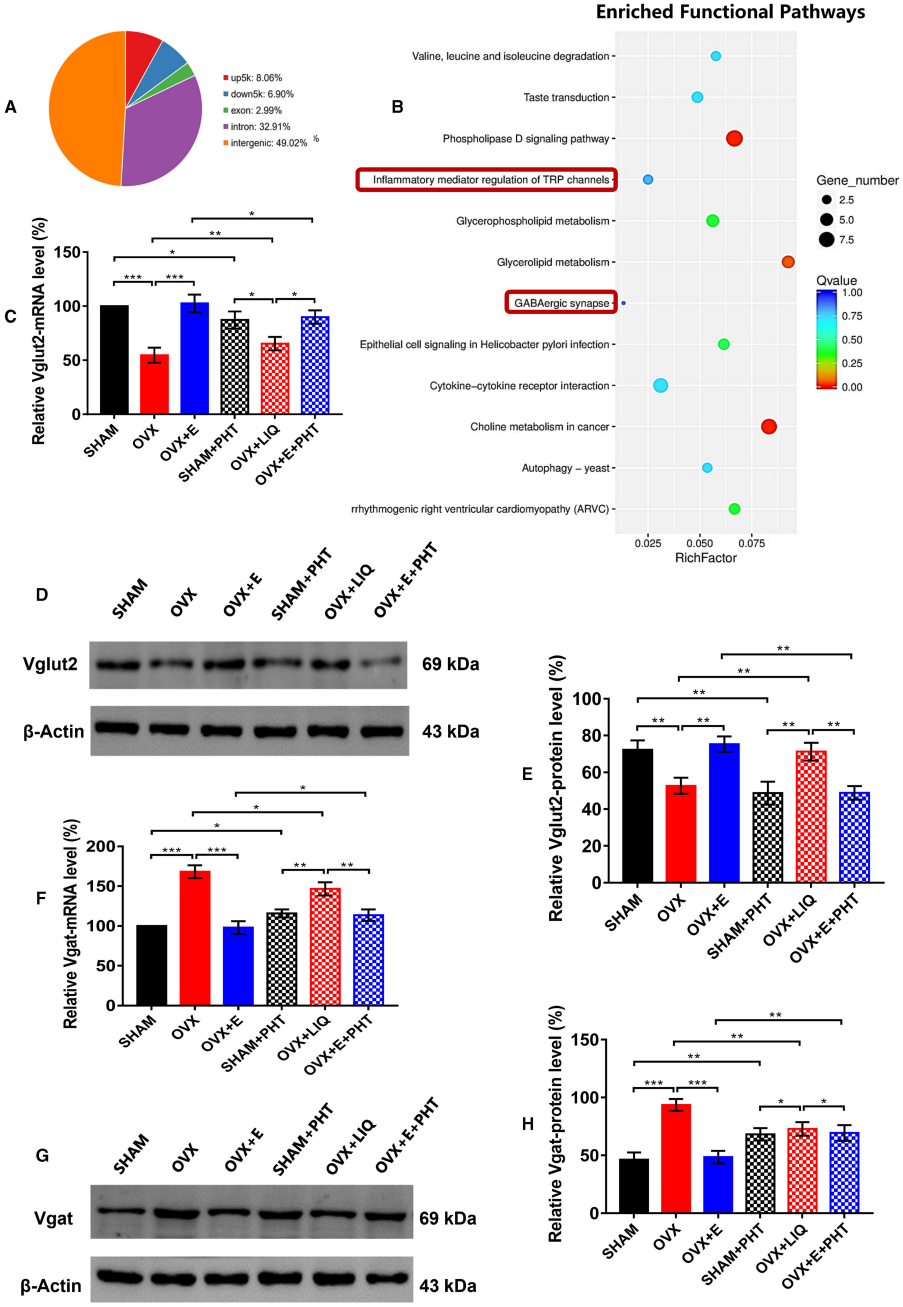
ERβ regulated Vglut2 and Vgat as a transcription factor. **(A)** Distribution of peaks pulled down by ERβ. **(B)** Pathways enriched by the peaks pulled down by ERβ. The red boxes indicate GABAergic synapse and inflammatory mediators of TRP channels. **(C)** Relative mRNA levels of Vglut2 (Vglut2/β-Actin) before and after the injection of agonists and inhibitors of ERβ; *n* = 5. **(D)** Immunoblots of Vglut2 (69 kDa) and β-Actin (43 kDa) before and after the injection of agonists and inhibitors of ERβ. **(E)** Relative protein levels of Vglut2 (Vglut2/β-Actin) before and after the injection of agonists and inhibitors of ERβ; *n* = 5. **(F)** Relative mRNA levels of Vgat (Vgat/β-Actin) before and after the injection of agonists and inhibitors of ERβ; *n* = 5. **(G)** Immunoblots of Vgat (69 kDa) and β-Actin (43 kDa) before and after the injection of agonists and inhibitors of ERβ. **(H)** Relative protein levels of Vgat (Vgat/β-Actin) before and after the injection of agonists and inhibitors of ERβ; *n* = 5. ERβ, estrogen receptor β; up5k, peaks located within the 5,000 bp upstream of the transcription start site; down5k, peaks located within the 5,000 bp downstream of the transcription start site; LIQ, Liquiritigenin; PHT, PHTPP; TRP, transient receptor potential.

In the published article, there were errors in the text. The abbreviation for ERα antagonist, AZD, was misspelled as ZAD due to a clerical error.

In the section, **Results**, *ER*α *regulated Vglut2 and vgat as a transcription factor*, Paragraph 2. This sentence previously stated:

“After injection of ERα agonist into POA of OVX group, the expression of mRNA and protein of Vglut2 increased significantly (90.36 ± 4.69 vs 43.4 ± 3.87, p < 0.001; 73.92 ± 4.85 vs 40.20 ± 3.46, p < 0.001; respectively), which was significantly higher than that in SHAM + AZD (p < 0.05) and OVX + E + ZAD (p < 0.05) groups, while there was no significant difference between SHAM + AZD and OVX + E + ZAD groups (Figures 9C–E).”

The corrected sentence appears below:

“After injection of ERα agonist into POA of OVX group, the expression of mRNA and protein of Vglut2 increased significantly (90.36 ± 4.69 vs. 43.4 ± 3.87, *p* < 0.001; 73.92 ± 4.85 vs. 40.20 ± 3.46, *p* < 0.001; respectively), which was significantly higher than that in SHAM + AZD (*p* < 0.05) and OVX + E + AZD (*p* < 0.05) groups, while there was no significant difference between SHAM + AZD and OVX + E + AZD groups (Figures 9C–E).”

In **Results**, *ER*α *regulated Vglut2 and vgat as a transcription factor*, Paragraph 3, the sentence previously stated:

“After injection of ERα agonist into POA of OVX group, the expression of mRNA and protein of Vgat decreased significantly (131.56 ± 8.14 vs 179.2 ± 7.94, p < 0.001; 66.36 ± 4.62 vs 87.72 ± 5.15, p < 0.001; respectively), and there was no significant difference between SHAM + AZD, OVX + PPT and OVX + E + ZAD groups (Figures 9F–H).”

The corrected sentence appears below:

“After injection of ERα agonist into POA of OVX group, the expression of mRNA and protein of Vgat decreased significantly (131.56 ± 8.14 vs. 179.2 ± 7.94, *p* < 0.001; 66.36 ± 4.62 vs. 87.72 ± 5.15, *p* < 0.001; respectively), and there was no significant difference between SHAM + AZD, OVX + PPT and OVX + E + AZD groups (Figures 9F–H).”

In **Results**, *ER*β *regulated Vglut2 and Vgat as a transcription factor*, Paragraph 2, the sentence previously stated:

“After injection of ERβ agonist into POA of OVX group, the expression of mRNA and protein of Vglut2 increased significantly (65.26 ± 6.25 vs. 54.61 ± 6.92, p < 0.01; 71.25 ± 4.85 vs, 52.64 ± 4.36, p < 0.01, respectively), which was significantly higher than that in SHAM + AZD (mRNA: p < 0.05; protein: p < 0.01) and OVX + E + ZAD (mRNA: p < 0.05; protein: p < 0.01) groups, while there was no significant difference between SHAM + AZD and OVX + E + ZAD groups (Figures 10C–E).”

The corrected sentence appears below:

“After injection of ERβ agonist into POA of OVX group, the expression of mRNA and protein of Vglut2 increased significantly (65.26 ± 6.25 vs. 54.61 ± 6.92, *p* < 0.01; 71.25 ± 4.85 vs, 52.64 ± 4.36, *p* < 0.01, respectively), which was significantly higher than that in SHAM + PHT (mRNA: *p* < 0.05; protein: *p* < 0.01) and OVX + E + PHT (mRNA: *p* < 0.05; protein: *p* < 0.01) groups, while there was no significant difference between SHAM + PHT and OVX + E + PHT groups (Figures 10C–E).”

In **Results**, *ER*β *regulated Vglut2 and Vgat as a transcription factor*, Paragraph 3, the sentence previously stated:

“After injection of ERβ agonist into POA of OVX group, the expression of mRNA and protein of Vgat decreased significantly (146.56 ± 8.14 vs. 168.26 ± 7.94, p < 0.05; 72.86 ± 5.81 vs. 93.5 ± 5.21, p < 0.01, respectively), which was still significantly higher than that in SHAM + AZD (mRNA: p < 0.01; protein: p < 0.05) and OVX + E + ZAD (mRNA: p < 0.01; protein: p < 0.05) groups, while there was no significant difference between SHAM + AZD and OVX + E + ZAD groups (Figures 10F–H).”

The corrected sentence appears below:

“After injection of ERβ agonist into POA of OVX group, the expression of mRNA and protein of Vgat decreased significantly (146.56 ± 8.14 vs. 168.26 ± 7.94, *p* < 0.05; 72.86 ± 5.81 vs. 93.5 ± 5.21, *p* < 0.01, respectively), which was still significantly higher than that in SHAM + PHT (mRNA: *p* < 0.01; protein: *p* < 0.05) and OVX + E + PHT (mRNA: *p* < 0.01; protein: *p* < 0.05) groups, while there was no significant difference between SHAM + PHT and OVX + E + PHT groups (Figures 10F–H).”

The authors apologize for these errors and state that these do not change the scientific conclusions of the article in any way. The original article has been updated.

