# Supplementary material for: Glutamatergic and GABAergic neurons in the preoptic area of the hypothalamus play key roles in menopausal hot flashes
Source: Front Aging Neurosci. 2022 Oct 14;14:993955. doi: 10.3389/fnagi.2022.993955 (PMC9614233; doi:10.3389/fnagi.2022.993955)
Supplement: Supplementary file 1 [file Data_Sheet_1.PDF]

Supplementary Material

1 Supplemental Tables

The data sets generated and analyzed during this study are available from the uploaded separately Supplemental Table S1-S4.

2 Supplemental Figures

Figure S1 The location of virus injection and the infection of glutamatergic and GABAergic neurons in the POA

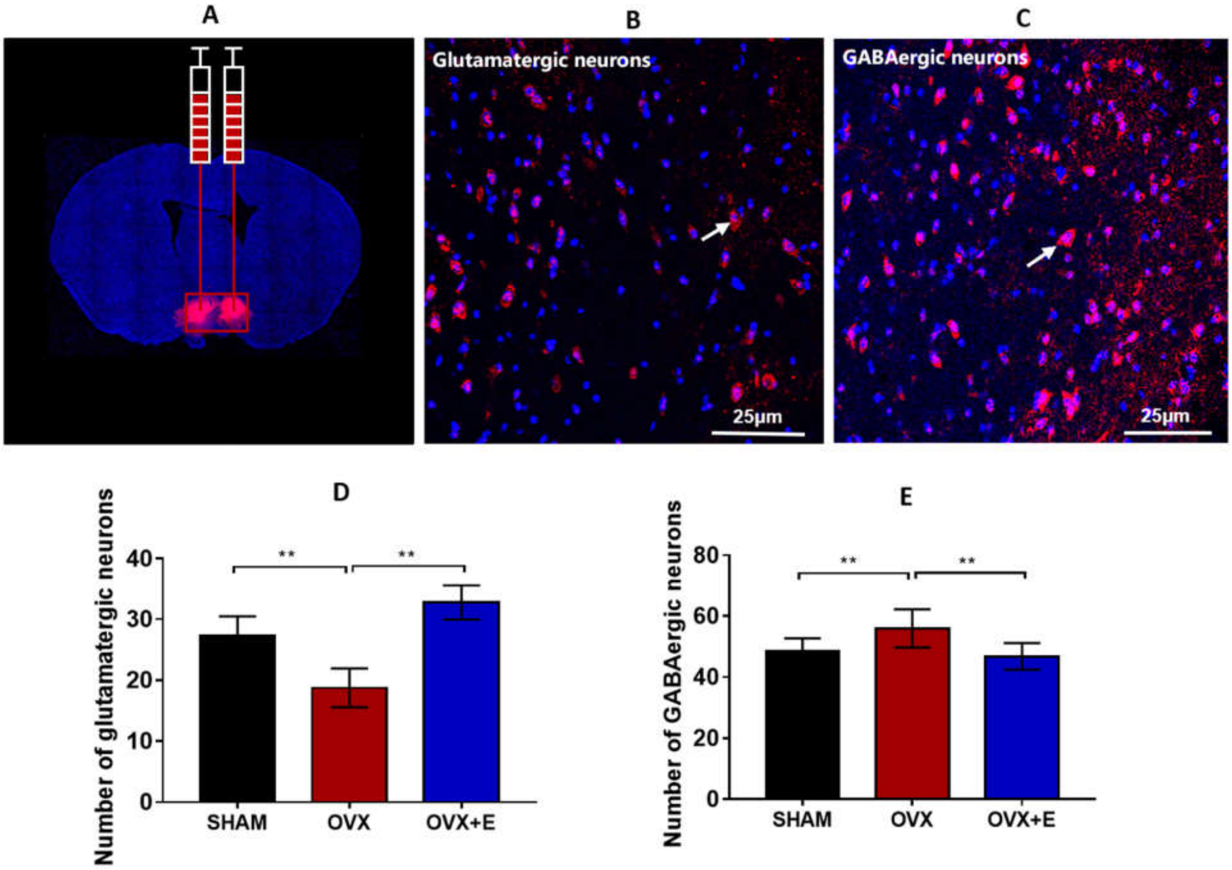

Figure S1. (A) The location of stereotactic injection of the virus into the POA of the hypothalamus (red). **(B)** Glutamatergic neurons infected by virus (red). **(C)** GABAergic neurons infected by virus (red). The blue was the nucleus labeled by DAPI, the arrow showed the representative neuron, bar= 25 μm. POA, preoptic area. **(D)** A statistical chart of the number of glutamatergic neurons infected by virus; n = 5.The number of glutamatergic neurons infected by virus in the POA was significantly lower in the OVX group than in the SHAM group (7.85 ± 0.42 vs 10.70 ± 0.89, p < 0·01), while they were significantly higher in the OVX+E group (10.85 ± 0.52 vs 7.85 ± 0.42, p < 0.01). There were no differences between the OVX+E and SHAM groups. **(E)** A statistical chart of the number of GABAergic neurons infected by virus; n=5.The number of GABAergic neurons infected by virus in the POA was significantly higher in the OVX group than in the SHAM group (7.85 ± 0.42 vs 10.70 ± 0.89, p < 0·01), while they were significantly lower in the OVX+E group (10.85 ± 0.52 vs 7.85 ± 0.42, p < 0.01). There were no differences between the OVX+E and SHAM groups. ANOVA was conducted to compare the outcomes among the three groups, and pairwise comparisons between the groups were conducted using LSD post hoc tests. The data are represented as the mean ± SD. *P < 0·05, **P < 0·01, ***P < 0·001. ANOVA, one-way analysis of variance; LSD, least significant

difference; OVX, ovariectomy with a vehicle; OVX+E, ovariectomy with estrogen; POA, preoptic area; SD, standard deviation; SHAM, sham surgery with a vehicle.

Figure S2 TST and BST of rats before virus injection and after injection of control virus

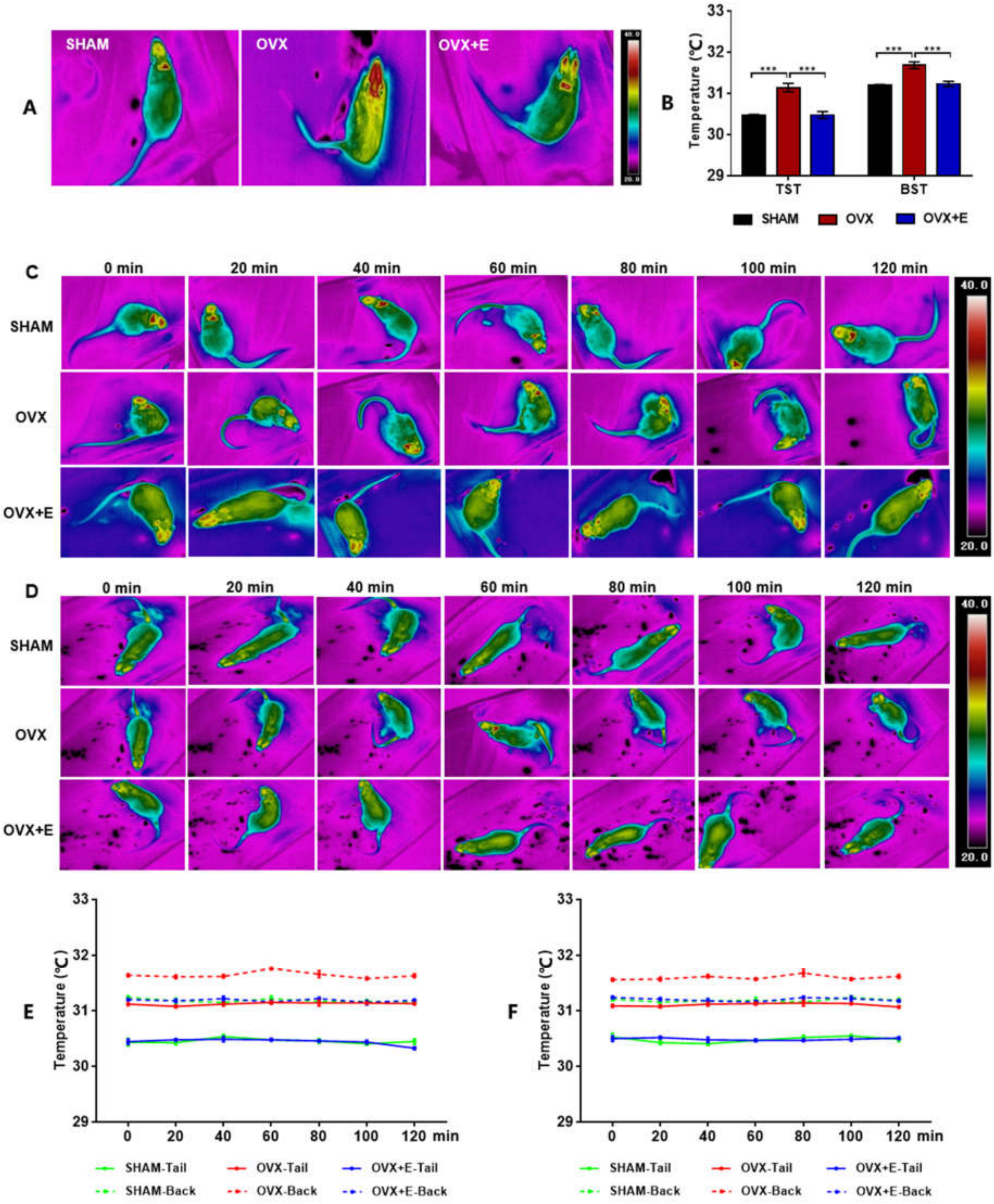

Figure S2. **(A)** Thermal imaging of rats before virus injection. **(B)** The statistical graph of the TST and BST; n = 5. **(C)** TST and BST of rats after Vglut2-control virus injection in the POA. **(D)** TST and BST of rats after Vgat-control virus injection in the POA. **(E)** Line chart of the TST and BST of rats after Vglut2-control virus injection every 20 min; n = 5. **(F)** Line chart of the TST and BST of

rats after Vgat-control virus injection every 20 min; n = 5. A TST and BST of rats after Vglut2-control virus injection in the POA; n = 5. NOVA was conducted to compare the outcomes among the three groups, and pairwise comparisons between the groups were conducted using LSD post hoc tests. The data are represented as the mean ± SD. *P < 0·05, **P < 0·01, ***P < 0·001. ANOVA, one-way analysis of variance; BST, back skin temperature; LSD, least significant difference; OVX, ovariectomy with a vehicle; OVX+E, ovariectomy with estrogen; POA, preoptic area; SD, standard deviation; SHAM, sham surgery with a vehicle; TST, tail skin temperature.
